# Genomic islands in *Pseudomonas* encode modular hotspots of defence and anti-defence systems

**DOI:** 10.1093/nargab/lqaf148

**Published:** 2025-11-19

**Authors:** Stephen R Garrett, Samantha K Tucker, Vojtech Pavelka, Andrew J Roe, Giuseppina Mariano

**Affiliations:** Michael DeGroote Institute for Infectious Disease Research, Department of Biochemistry and Biomedical Sciences, McMaster University, Hamilton, ON L8S 4K1, Canada; School of Infection and Immunity, 120 University Place, University of Glasgow, Glasgow G12 8TA, United Kingdom; School of Infection and Immunity, 120 University Place, University of Glasgow, Glasgow G12 8TA, United Kingdom; School of Infection and Immunity, 120 University Place, University of Glasgow, Glasgow G12 8TA, United Kingdom; School of Infection and Immunity, 120 University Place, University of Glasgow, Glasgow G12 8TA, United Kingdom

## Abstract

Bacteria use diverse defence systems to resist phage predation, many of which cluster within mobile genetic elements (MGEs) and defence islands. In *Pseudomonas aeruginosa*, genomic and pathogenicity islands—such as the pathogenicity islands (PAPI), genomic islands (PAGI), and Liverpool epidemic strain islands (LESGI)—have been linked to virulence and adaptation, but their contribution to the organization and spread of defence systems remains unexplored. Here, we show that these islands serve as hubs for the assembly and spread of defence systems, revealing an underappreciated role in shaping the bacterium’s antiviral arsenal. We identify 11 conserved hotspots that encode defence and anti-defence genes, but rarely co-occur with virulence factors, resistance genes, or interbacterial competition modules. The frequent co-occurrence of defence and anti-defence genes within these loci points to an ongoing, intense molecular arms race between bacteria, MGEs, and lytic phages. Notably, these hotspots are found beyond their original island contexts, appearing across diverse *Pseudomonas* species and, in some cases, other genera. Together, our findings expand the known bacterial immunity landscape in *P. aeruginosa*, redefine the roles of these islands as defence and anti-defence reservoirs, and establish a framework for scalable discovery and annotation of novel defence and anti-defence systems in bacterial genomes.

## Introduction

Bacteriophages (phages) and other mobile genetic elements (MGEs) continuous predation against bacteria led to the evolution of hundreds of diverse defence strategies [1, 2]. These systems include classical defences such as restriction-modification (RM) and CRISPR–Cas, as well as more recently identified systems like BREX (bacteriophage exclusion), DISARM (defence island system associated with restriction–modification), Hachiman, Shield, Zorya, and many others [3–12]. Such systems are often encoded together within defence islands—horizontally acquired loci that frequently accumulate multiple defence systems alongside other adaptive traits, typically integrated into bacterial chromosomes [13–16]. In other instances, defence systems accumulate on MGEs, such as plasmids, prophages, and integrative conjugative elements (ICE). When carried on MGEs, defence systems are often encoded within more narrowly defined variable hotspots. These represent spatially confined loci, often bound by conserved structural or constitutive genes of the MGE (e.g. integrases, terminases, or replication proteins) [10, 13, 14, 17, 18]. For example, in P2-like phages, a conserved replication endonuclease and portal protein demarcate a variable region that frequently encodes a diverse array of defences [10].

In response to the evolution of defence systems, many MGEs have acquired counter-measures that favour either evasion or disablement of bacterial defence strategies. For example, several MGEs encode anti-restriction modification (anti-RM) proteins, such as the Ocr protein of phage T7. Ocr acts as a DNA mimic that binds and inhibits restrictions enzymes [19, 20]. Similarly, some phages and other MGEs carry anti-CRISPR (Acr) proteins [21, 22], which inhibit CRISPR–Cas effector complexes, preventing DNA cleavage. Several phage species can also deploy sponge proteins that sequester signalling molecules that drive the activation of bacterial defence systems. Well-characterized examples are the Acb and Tad families, which bind cyclic oligonucleotides to block activation of CBASS and Thoeris systems respectively, effectively preventing the initiation of abortive infection [23–25]. Given that MGEs must overcome host defences to establish themselves within bacterial populations, it is unsurprising that anti-defence proteins are frequently encoded not only by lytic phages but also by MGEs [2, 26, 27].

The plasticity of the *Pseudomonas aeruginosa* pangenome is driven by the acquisition of diverse MGEs and large, horizontally acquired genomic islands (GIs). These are distinct chromosomal regions introduced by horizontal gene transfer, frequently inserted near tRNA genes and commonly carrying mobility, defence, or virulence genes [28]. Over time, systematic analyses have identified at least two PAPI (*Pseudomonas aeruginosa* pathogenicity islands), 17 LESGI (Liverpool epidemic strain genomic islands), and 11 PAGI (*Pseudomonas aeruginosa* genomic islands) distributed across *P. aeruginosa* genomes [29–33].

PAPI-X, PAGI-X, and LESGI-X islands have been studied in the context of conferring virulence and antibiotic resistance traits [28, 32]. For example, the PA14 strain contains two pathogenicity islands, PAPI-1 and PAPI-2, which encode multiple virulence factors, such as the PAPI-2 ExoU toxin [34]. Other islands of the PAGI-X and LESGI-X family instead carry metabolic, transport, and antibiotic resistance genes [29, 30, 35]. Typically, GIs derive from MGEs and frequently contain remnants of prophages, transposons or ICEs [31]. For example, PAPI-1 and PAGI-5 represent intact integrative conjugative elements (ICEs) with mobility potential [31, 36]. In contrast, *P. aeruginosa* PAPI-2 island derives from the pKLC102-like ICE and carries an integrase; nevertheless, it lacks a conjugation machinery, thus not representing a canonical ICE [32, 37]. Recent studies have further classified LESGI-3, PAGI-2, and PAGI-3 as ICE-like elements based on their integrase content and partial synteny with other ICEs [33, 34].

Although PAPI-X, PAGI-X, and LESGI-X islands have been extensively studied in *P. aeruginosa*, the prevalence of defence/anti-defence genes on these elements has not been extensively investigated. To date, only two defence hotspots, cDHS1 and cDHS2, have been described in *P. aeruginosa* [38], and neither was significantly associated with known PAPI-X, PAGI-X, or LESGI-X regions. Furthermore, recent studies have shown that certain ICE-like elements in *P. aeruginosa*, including PAGI-2, PAGI-3, and LESGI-3, can encode defence systems such as CBASS, and that novel PAGI-like elements in clinical isolates carry anti-CRISPR proteins [26, 35]. However, these observations have focused on specific systems and islands, without a systematic exploration of defence system distribution across the broader PAPI-X, PAGI-X, and LESGI-X families.

In this study, we report a comprehensive analysis of defence and anti-defence systems within *P. aeruginosa* GIs, focusing on the PAPI-X, PAGI-X, and LESGI-X families. Using comparative genomics, we identify numerous GIs enriched in defence genes and delineate eleven hotspots (Table 1)—discrete and delimited loci that harbour diverse immunity or anti-defence systems.

**Table 1.** Overview of hotspots identified across *P. aeruginosa* genomic islands

Island family	Hotspot identified
PAPI-1	GI-Hotspot 1, GI-Hotspot 2, GI-Hotspot 3
PAPI-2	No discrete hotspots; defence systems span the full island
PAGI-1	GI-Hotspot 2, GI-Hotspot 4, GI-Hotspot 5, GI-Hotspot 6, GI-Hotspot 7
PAGI-2	GI-Hotspot 8
PAGI-3	GI-Hotspot 8
PAGI-4	None detected
PAGI-5	None detected
PAGI-6	None detected
PAGI-7	None detected
PAGI-8	None detected
PAGI-9	None detected
PAGI-10	None detected
PAGI-11	None detected
LESGI-1	GI-Hotspot 9
LESGI-2	None detected
LESGI-3	GI-Hotspot 8
LESGI-4	None detected
LESGI-5	None detected
LESGI-6	None detected
LESGI-7	None detected
LESGI-8	None detected
LESGI-9	None detected
LESGI-10	None detected
LESGI-11	GI-Hotspot 10, GI-Hotspot 11
LESGI-12	None detected
LESGI-13	None detected
LESGI-14	None detected
LESGI-15	None detected
LESGI-16	None detected
LESGI-17	None detected

By defining the conserved boundaries of these hotspots, we not only map their internal organization but also demonstrate that these loci occur beyond canonical PAPI-X, PAGI-X, and LESGI-X elements and in some cases, in species other than *P. aeruginosa*. This highlights the scalability of our approach and establishes the hotspot boundaries identified here as a powerful resource for future discovery of novel defence and anti-defence systems in *P. aeruginosa* and related species.

## Materials and methods

### Identification of PAPI-X, PAGI-X, and LESGI-X homologues

To identify homologues of the PAPI-X, PAGI-X, and LESGI-X islands, BLASTn searches were performed [39]. For each BLASTn search, the canonical representative sequence of each genomic island was used as the query. The corresponding query sequences and their genomic coordinates are provided in Supplementary Table S36. Searches were performed on a nucleotide level against a RefSeq database. Hits were filtered based on a query and subject coverage of 50%–100% and a minimum sequence identity of 30%. Custom Python scripts (https://github.com/GM110Z/PAPI-islands-analysis/tree/main and https://github.com/GM110Z/PAPI-islands-analysis/releases/tag/gi-hotspots) were subsequently used to measure the size of each hit and to further filter the results, retaining only those whose size differed by no more than 30% from the canonical size of the representative island. These thresholds were chosen after testing multiple parameter combinations, as they minimized spurious overlaps between islands while retaining true homologues, taking into account the modular similarity common among genomic islands.

The resulting hits were used to construct the island datasets analysed in this study (Supplementary Table S1). Subsequently, the Python library pandas was used to compare the filtered BLASTn results and determine the overlap between different islands. Matplotlib was then used to plot the results [67, 68]. Proteome pairwise comparisons were performed using LoVis4u [17] and plotted with a chord diagram through R. Nucleotide alignment of PAPI-X, PAGI-X, and LESGI-X representative sequences were obtained through Clinker [69].

### Proteins function predictions in *Pseudomonas* GI islands

To initially characterize the content of *Pseudomonas* genomic islands, protein sequences encoded on each island were analysed using the Virulence Factor Database (VFDB) and a database of T6SS effectors, from SecreT6 [40–42]. Searches against each database were performed using PSI-BLAST, with hits filtered by an *e*-value < 0.01 and a query/subject coverage of at least 50% [40–42]. Proteins were classified into virulence factor categories based on the definitions provided by the VFDB (https://www.mgc.ac.cn/VFs/main.htm).

The defence systems content of each island was predicted using PADLOC v2.0.0 with PADLOC database v2.0.0 and DefenseFinder v1.3.0 [46, 70]. Anti-defence proteins were predicted using DefenseFinder v1.3.0 with the -a flag [2].

### Identification and extraction of novel defence hotspots

Only islands with ≥5 distinct defence system subtypes (as annotated by PADLOC/DefenseFinder) were selected for hotspot identification. This cutoff was chosen to avoid overestimating hotspots from random co-occurrence of 1–2 systems and to ensure that identified regions reflect genuine loci of defence enrichment.

To identify defence/anti-defence hotspots, 25 kb regions upstream and downstream of systems predicted by PADLOC v2.0.0 and DefenseFinder v1.3.0 were retrieved using bedtools, faidx, and the Entrez efetch suite [71–73]. The Biopython library SeqIO was additionally used to convert GenBank files to protein fasta files [74]. Protein sequences were clustered using MMseqs2 linclust with the following parameters: –cov-mode 0, -c 0.5, and –min-seq-id 0.6 [75]. To identify hotspot boundaries, protein clusters were paired based on similar frequencies of occurrence and their positioning on opposite sides of known defence systems, in close proximity. For this purpose, custom scripts based on the Python libraries pandas, numpy, defaultdict, and SeqIO were used [74] (https://github.com/GM110Z/PAPI-islands-analysis/tree/main and https://github.com/GM110Z/PAPI-islands-analysis/releases/tag/gi-hotspots). All hotspot calling was performed separately within each genomic-island family (e.g. PAGI-1 and PAPI-1), using only the local enrichment of defence/anti-defence annotations relative to that family’s background.

Once hotspot boundary candidates were identified, 100 homologous proteins for each boundary were collected through BLASTp and aligned through MUSCLE v5.1 [76]. Alignments were then used to build HMM models [77]. Finally, a custom script was used to identify and extract genomic regions where the HMM models were found to colocalize and define hotspot regions with a maximum length of 40 kb (https://github.com/GM110Z/PAPI-islands-analysis/tree/main and https://github.com/GM110Z/PAPI-islands-analysis/releases/tag/gi-hotspots). For each hotspot boundary pair, we restricted extraction to the island family where that hotspot was first identified (e.g. candidates from PAGI-1 were applied only to PAGI-1 sequences)

Defence systems were identified with PADLOC v2.0.0 with PADLOC database v2.0.0 and DefenseFinder v1.3.0 [46, 70]. Anti-defence systems were identified with DefenseFinder v1.3.0. AMRFinder v3.11.26 was used to detect antibiotic resistance genes. For hotspot content not defined by these methods, we performed hmmscan searches against the PFAM database. Extracted regions were scored as defence/anti-defence hotspots if they encompassed distinct defence systems in at least 10% of instances [2, 45, 46, 70, 77, 78].

### Defence system distribution analysis

Predicted defence systems identified were mapped to genomic coordinates and assigned to either specific GI-Hotspots 1–11 or to Elsewhere (any locus outside these hotspots, including other defence islands, prophages, or MGEs). Defence systems prediction output was de-duplicated at the system level to account for multiple components of the same system in a given locus. For each defence system type, we calculated its total occurrence across the genome and the number of occurrences within each hotspot. We then expressed hotspot contributions as fractions of the total genomic occurrences. For visualization purposes, the top 50 most frequent systems (by total occurrence across all genomes in the dataset) were retained for plotting. Custom scripts available at https://github.com/GM110Z/PAPI-islands-analysis/releases/tag/gi-hotspots

### Anti-defence system distribution analysis

Anti-defence systems predicted with DefenseFinder were analysed in the same manner as above, except that loci were defined by subtype–protein pairs. Each subtype–protein pair was matched between the genome-wide dataset and the hotspot-specific datasets, and per-pair counts were obtained for each hotspot and for Elsewhere. Fractional contributions were calculated relative to the total genomic count per subtype–protein pair. For visualization purposes, the top 50 most frequent systems (by total occurrence across all genomes in the dataset) were retained for plotting. Custom scripts available at https://github.com/GM110Z/PAPI-islands-analysis/releases/tag/gi-hotspots.

### Identification of GI-Hotspots 1–11 across all bacterial genomes in NCBI

To expand our search beyond the BLASTn-derived database of genomic islands—particularly given that nucleotide identity can be low among mobile elements and genomic islands—we used cblaster v1.3.12 [48] with the following parameters: -min id = 30 and -min cov = 50, to investigate whether GI-Hotspots 1–11 are present in other *Pseudomonas* strains and across diverse bacterial species. Searches were performed against a Refseq protein database using representative hotspot boundary proteins as queries (Supplementary Tables S12, S18, S23, S27, and S33). Unlike BLASTn, which may miss remote homologues due to sequence divergence at the nucleotide level, cblaster performs protein-level homology searches using BLASTp and detects clusters of co-localized proteins based on a defined intergenic distance threshold [48].

### Phylogenetic analysis

A whole-genome distance tree was generated with Mashtree v1.4.6 using a 5000 k-mer sketch size [79]. The resulting Newick tree was midpoint-rooted, ladderized, and plotted in Rstudio using ggtree and ggplot2 [80, 81]. A binary presence–absence matrix of genomic islands was aligned to the tip order using gheatmap, missing values were set to zero, and columns were ordered by decreasing prevalence.

To control for clonal overrepresentation, we clustered genomes by pairwise Mash distance at *d* ≤ 0.001 and genomes grouped in clusters based on the computed distances. We then collapsed the genome-by-island presence/absence matrix to the cluster level by logical OR (a cluster scored “present” if any member carried the island). Co-occurrence was recomputed on this cluster-level matrix using Fisher’s exact test on 2 × 2 contingency tables (Haldane–Anscombe correction, +0.5 to each cell), with Benjamini–Hochberg FDR control to obtain *q*-values [82, 83]. To summarize robustness, we compared full-set versus cluster-level results using: (i) the fraction of originally significant pairs retained, (ii) Jaccard overlap between significant-pair sets, (iii) direction concordance of effects (OR > 1 versus OR < 1), (iv) Spearman correlation of log₂(OR), and (v) the median absolute change in effect size (|Δlog₂OR|). Unless stated, significance was defined as *q* < 0.05. The redundancy analyses are reported in Supplementary Table S4.

### Statistical assessment of defence system enrichment

To evaluate if the enrichment of defence and anti-defence systems in each hotspot relative to a background expectation of 5% is statistically significant, we first calculated the observed proportion of genomes with the system as *p̂* = *k*/*n*, where *k* is the number of genomes encoding a defence or anti-defence system, and *n* is the total genomes carrying that hotspot. Fold enrichment was calculated as *p̂*/0.05. Statistical significance was assessed using a one-sided binomial test under the null hypothesis : H_0_:*p*= 0.05, with *P*-values computed as *P*(*X* ≥ *k*∣*X*∼Binomial(*n*,0.05)), where *P* denotes the probability of observing *k* or more genomes that carry a defence or anti-defence system out of *n* total genomes, assuming a background/random localization of defence systems in any region of a genome to be below 5%. The threshold of 5% occupancy was guided by the distribution reported in Hochhauser *et al.*, 2023 [14], where hotspot occupancy ranged from 0.23% to 97.26%. In this dataset, the median occupancy was ∼7.6%, and ∼90% of hotspots showed an occupancy ≥5%. Thus, our cutoff excludes the extreme low-occupancy tail while retaining the vast majority of biologically meaningful sites and minimizes false positives in divergent genomic islands. Additionally, Bayesian posterior probabilities that the true proportion exceeds 5%, *P*(*p*> 0.05∣ *k,n*), were calculated assuming a uniform Beta (1,1) prior. The posterior distribution is Beta-distributed as Beta(*k* + 1, *n* − *k* + 1) and the posterior probability was calculated as *P*(*P*> 0.05) = 1 − *F*_Beta_(0.05;*k* + 1,*n* − *k* + 1).

All calculations were performed using a custom Python script, which is available at https://github.com/GM110Z/PAPI-islands-analysis/tree/main/Statistics and https://github.com/GM110Z/PAPI-islands-analysis/releases/tag/gi-hotspots.

## Results

### *Pseudomonas aeruginosa* pathogenicity and genomic islands are a rich source of defence systems

To collect and classify *P. aeruginosa* genomic islands (GIs) we used the representative sequence of each PAGI-X, PAPI-X, and LESGI-X available on NCBI using BLASTn against a RefSeq database (Materials and methods) [29, 30, 32, 39]. Hits were filtered based on a query and subject coverage of 50%–100%. Additionally, for each island, hits were retained only if their size differed by no more than >30% from the canonical size of the representative island [29, 30, 32, 39] (Materials and methods).

**Figure 1.**
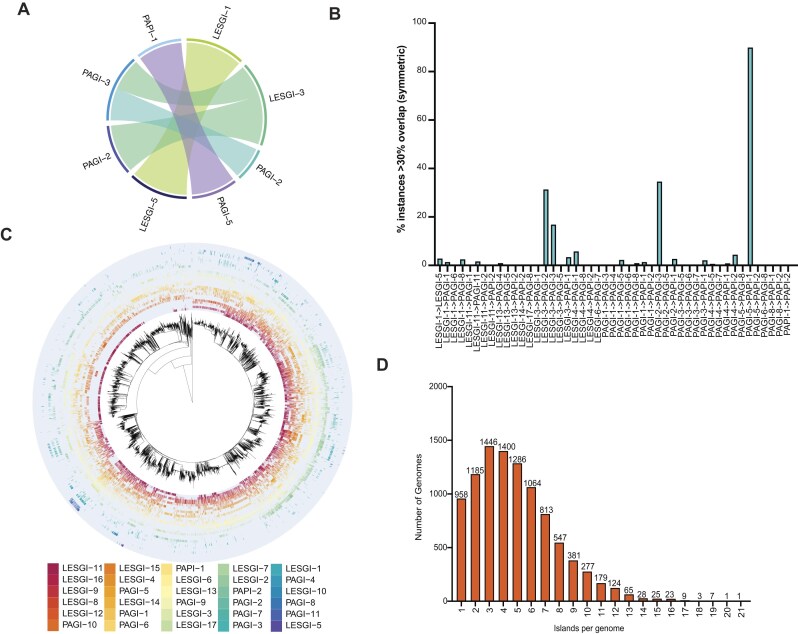
*Pseudomonas aeruginosa* genomic islands can carry a variable defence arsenal. (**A**) Chord diagram summarizing the proteomic-level comparisons between representative sequence from each PAPI-X, PAGI-X, and LESGI-X group. Each sector represents a genomic island representative. Chords between sectors indicate pairwise similarity relationships based on bidirectional proteome comparison. Only pairs with mutual similarity scores ≥ 0.4 are shown. The width of each chord is proportional to the similarity score, highlighting stronger connections with thicker links. (**B**) Bars show the symmetric fraction of instances where two island types overlap by >30% of the smaller island. For each island pair, we first counted how many unique instances of Island A overlap >30% with at least one instance of Island B, and vice versa. Percentages were then normalized by the total number of instances of each island type, and the symmetric value shown here corresponds to the smaller of the two percentages. Thus, a bar of 10% means that at least 10% of both Island A and Island B instances exhibit substantial (>30%) mutual overlap within the same assembly/contig. (**C**) Neighbour-joining tree generated with mashtree from Mash distances (sketch size 5000) and plotted in a circular layout (midpoint-rooted, ladderized). The outer ring shows a presence–absence heatmap of genomic islands per genome (rows follow tip order): absence is light grey; presence is encoded with a distinct colour per island, as per legend. Island columns are ordered by decreasing prevalence across genomes. (**D**) Distribution of genomic island richness across bacterial assemblies. Bar plot showing the number of genomes carrying various combinations (1, 2, 3… up to 17) of distinct genomic islands. Island abundance was calculated as the total number of unique islands detected in each genome, using NCBI assembly identifiers to account for islands occurring on different contigs. Values are based on the binary presence/absence matrix.

Given previous reports of sequence similarity between PAPI-1 and PAGI-5 and the general mosaic structure of PAGI-X, PAPI-X, and LESGI-X elements [30, 32, 37], we first assessed the extent of nucleotide sequence and proteome-level similarity among representative sequences for each group (Fig. 1A and Supplementary Fig. S1A). This analysis confirmed the high degree of nucleotide and proteome overlap between PAGI-5 and PAPI-1. Additional similarities were observed between LESGI-1 and LESGI-5, as well as among PAGI-2, LESGI-2, and LESGI-3. Although these latter islands share conserved regions, each also harbours unique segments spanning at least 30% of their total length (Fig. 1A; Supplementary Fig. S1A and Supplementary Tables S1 and S2). PAGI-1 and LESGI-4 similarly exhibit substantial sequence and proteome homology; however, both contain an additional distinct region exclusive to each island (Fig. 1A; Supplementary Fig. S1A and Supplementary Tables S1 and S2).

To ensure robust group definitions, we next analysed all sequences from each island group obtained using BLASTn, comparing genomic coordinates to assess potential redundancy. PAGI-2 overlaps with PAGI-3 in 34.5% of instances; 31.3% of PAGI-2 regions overlap with LESGI-3, whereas 16% of LESGI-3 overlap with PAGI-3 (Fig. 1B). Conversely, PAPI-1 and PAGI-5 showed extensive redundancy, with >90% of identified islands overlapping in sequence coordinates (Fig. 1B and Supplementary Table S3). The remaining genomic islands groups did not exhibit substantial overlap, supporting that our filtering thresholds based on island size and percentage identity effectively distinguished between groups (Fig. 1B and Supplementary Table S3). Due to the high degree of similarity between PAPI-1 and PAGI-5, we treated them as a single group in subsequent analyses.

We next investigated the co-occurrence of multiple island types within individual genomes to assess patterns of island compatibility. The most frequent pairs are LESGI-11 + PAGI-10 (*n* = 90), LESGI-11 + LESGI-14 (*n* = 63), LESGI-11 + LESGI-15 (*n* = 53), and LESGI-11 + PAPI-2 (*n* = 39). Among triplets, the most common are LESGI-11 + LESGI-15 + PAGI-10 (*n* = 41), followed by LESGI-11 + PAGI-10 + PAPI-1 (*n* = 15) and LESGI-11 + LESGI-14 + LESGI-15 (*n* = 15). Larger combinations are rarer but present, for example, LESGI-11 + LESGI-15 + LESGI-17 + LESGI-6 + PAPI-2 (*n* = 30) (Supplementary Fig. S1C and Supplementary Table S4). To place these patterns in a phylogenetic context, we mapped LESGI-X/PAGI-X/PAPI-X presence/absence onto a whole-genome distance tree inferred with Mashtree (Fig. 1C). Two signatures were evident: (i) long, clade-bounded blocks in which island pairs switch state at internal nodes, indicating co-localization largely attributable to shared ancestry; and (ii) mosaic distributions of many single islands across phylogenetic splits, consistent with recurrent horizontal transfer and/or frequent loss, with systematic absences in specific clades suggesting host-range or compatibility constraints (Fig. 1C). Across the dataset, most genomes carried one to six distinct genomic islands, with only a minority harbouring larger combinations (Fig. 1D).

To assess whether these signatures were inflated by clonal redundancy, we collapsed near-identical strains into clusters using Mash (Materials and methods) and re-estimated co-occurrence on the cluster-level matrix. This approach shows 73.3% of significant pairs are retained (Jaccard = 0.631), effect directions are largely unchanged (88.4% concordant), and effect sizes agrees in rank (Spearman *r* = 0.841 for log₂(OR); median |Δlog₂OR| = 0.339, 95th = 1.166) (Supplementary Table S4). Consistent with this, the leading combinations remain enriched and significant at both levels, including LESGI-11 + PAGI-10, LESGI-11 + LESGI-14, LESGI-11 + LESGI-15, and LESGI-11 + PAPI-2; likewise the triplets LESGI-11 + LESGI-15 + PAGI-10, LESGI-11 + PAGI-10 + PAPI-1, and LESGI-11 + LESGI-14 + LESGI-15 (Supplementary Table S4). We also note that BLASTn retrieval differed across islands, with some loci returning markedly fewer hits, reflecting either genuinely narrower spread or biases in RefSeq composition. Such differences naturally reduce observed co-occurrences and should be considered when interpreting compatibility.

Next, to determine whether *Pseudomonas* GIs accommodate a variable cargo or are specialized for distinct types of bacterial conflict modules, we used PADLOC and DefenseFinder to predict defence systems, and PSI-BLAST to search the Virulence Factor Database (VFDB) and a database of Type VI Secretion System effectors from SecreT6 [40–42]. Under these parameters, we did not recover confident functional assignments for proteins encoded on PAGI-9, PAGI-10, PAGI-11, and LESGI-2, LESGI-8, LESGI-9, LESGI-12, LESGI-16. Battle *et al.* reported that PAGI-9 and PAGI-10 resemble Rhs elements but lack canonical VgrG/Hcp components, instead encoding divergent Rhs proteins [30]. Additional studies indicate that LESGI-8 encodes Type VI secretion system components; LESGI-9 harbours partial clusters comprising NADH dehydrogenases, methyltransferases, uncharacterized proteins, and a secreted protein; LESGI-12 carries a porin associated with antibiotic resistance; and LESGI-16 encodes multiple enzymes (isomerases, reductases, and nucleotidyl-transferases) as well as several transfer RNA (tRNA) genes [29]. Finally, a separate study reported that LESGI-2 carries a biosynthetic cluster for the antifungal molecule pyoluteorin [43].

Whilst we did not detect a direct correlation between the average size of each island group and the presence of defence proteins, virulence, or anti-bacterial effectors, we found that GI groups with the highest variability in size were more likely to carry these types of genes (Supplementary Fig. S1B).

**Figure 2.**
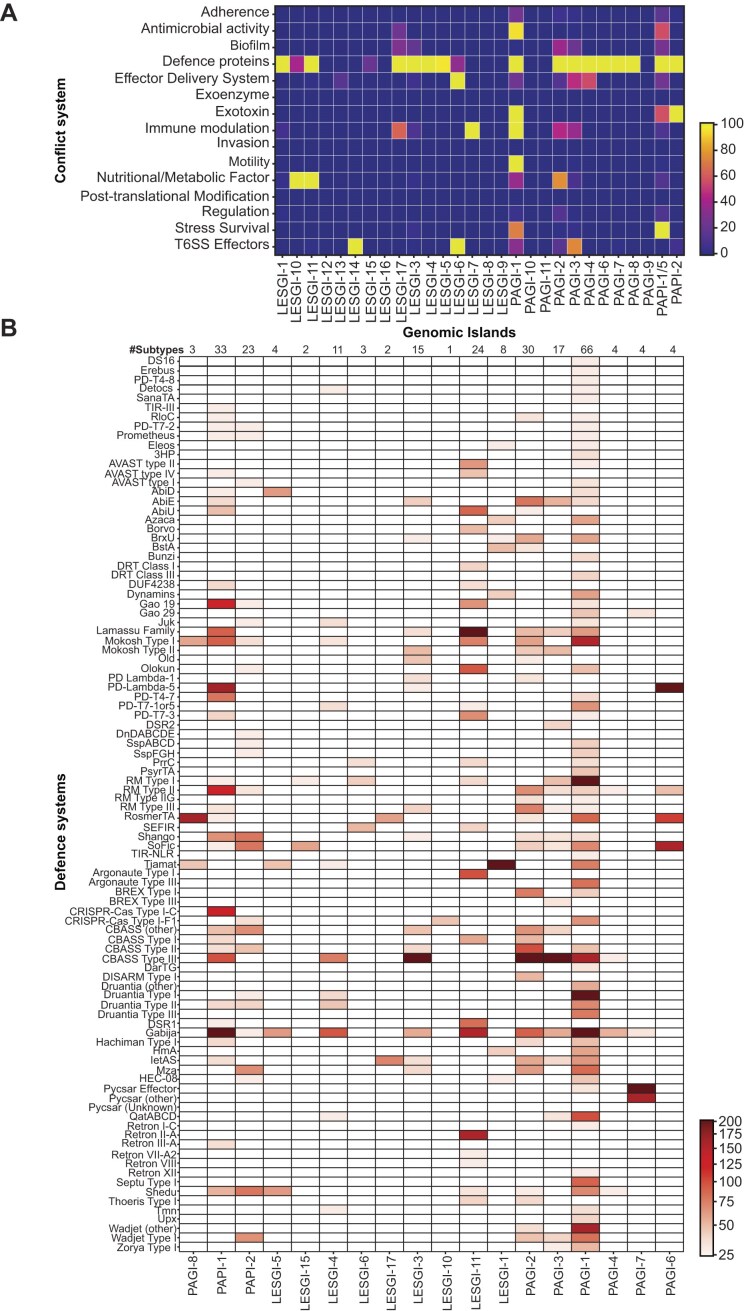
*Pseudomonas* genomic islands carry numerous defence and anti-defence systems. (**A**) Heatmap representing the presence and relative abundance of conflict-associated systems, including defence mechanisms, Type VI Secretion System (T6SS) components, and virulence factors (VFDB-annotated), across distinct GIs. Each row represents a different system type, while each column corresponds to an individual genomic island group. Data were visualized using the plasma colour scale. (**B**) Heatmap depicting the presence/absence patterns of defence systems across GIs that encode more than five distinct systems. Rows represent individual defence system types, while columns correspond to genomic islands. Presence is indicated by a gradient in red colour intensity proportional to the number of hits (logarithmically scaled using a power-normalized colour scale), whereas absences are masked and shown in white. Defence systems were predicted with PADLOC v.2.0.0 and DefenseFinder v1.3.0. The number of total defence systems found in each island is annotated at the top of each column.

Our analysis further revealed that, except for LESGI-7,8,9, 12, 13, 14, 16, PAGI-9, PAGI-10, and PAGI-11, the remaining GIs possess a rich arsenal of defence proteins, often outnumbering known virulence factors and anti-bacterial toxins (Fig. 2A and Supplementary Tables S5–S7). LESGI-6 and LESGI-14, while lacking a significant number of defence systems, frequently harbour T6SS effectors (Fig. 2A and Supplementary Table S7). LESGI-10 and LESGI-11 carry genes involved in secondary metabolite production, with LESGI-10 encoding genes for pyoverdine biosynthesis and LESGI-11 for phenazine synthesis (Fig. 2A and Supplementary Table S6). Additionally, in agreement with previous reports, PAGI-1 and PAPI-2 islands exhibit a high number of exotoxins, with ExoU predominantly represented in PAPI-2 and ExoA prevalent in PAGI-1 (Fig. 2A and Supplementary Table S6). Finally, PAPI-1 and PAGI-2 encode additional functional elements alongside defence proteins, including exotoxins, factors involved in secondary metabolite production, biofilm formation, and those that may provide a competitive advantage (Fig. 2A and Supplementary Table S7). In particular, PAPI-1 contains genes predicted to be mobility-associated, likely corresponding to a type IV pilus involved in its conjugative transfer (Fig. 2A and Supplementary Table S6) [31].

Having identified the prevalence of individual defence proteins, we next examined their native genomic context to determine whether they co-occurred within the same operon, formed part of a complete defence system and to assess their diversity across genomic islands. To identify GIs with the most diverse defence repertoires, we considered those encoding at least five distinct defence systems, to ensure that only highly variable defence hotspots were included in the analysis (Fig. 2B). We found that PAPI-1/PAGI-5, PAPI-2, PAGI-1, PAGI-2, PAGI-3, LESGI-1, LESGI-3, and LESGI-11 harbour the highest diversity of defence systems (Fig. 2B). These were therefore selected for further analysis to identify potential novel hotspots.

### PAPI-1 islands carry three distinct hotspots

PAPI-1 and PAGI-5 encode one of the highest diversities of defence proteins (Fig. 2A). To assess whether these systems are randomly distributed within the PAPI-1/PAGI-5 islands or concentrated in specific hotspots, we first clustered the proteins encoded on these regions. The identified protein clusters were then paired based on their similar frequencies of occurrence and proximity to known defence systems. For each cluster pair, Hidden Markov Models (HMMs) were generated to extract candidate hotspots. Cluster pairs were considered to define boundaries of a hotspot if, following extraction, they encompassed distinct defence or anti-defence systems in at least 10% of identified PAPI-1/PAGI-5 instances (Table 1 and Supplementary Table S8).

**Figure 3.**
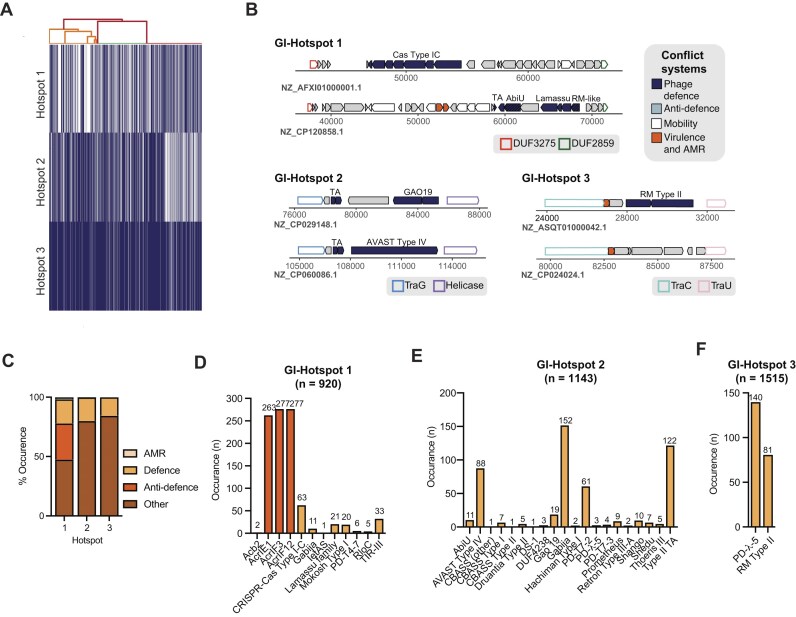
PAPI-1 pathogenicity island is a rich reservoir of defence and anti-defence systems. (**A**) The presence–absence matrix visualizing the distribution of the three hotspots—GI-Hotspots 1, 2, and 3—found in PAPI-1 instances identified by BLASTn. Each row corresponds to a hotspot, while each column represents a distinct genome (nucleotide accession). Presence is indicated in dark blue and absence in white. The corresponding dendrogram groups genomes based on similarity in hotspot content, using Ward’s linkage and Euclidean distance for hierarchical clustering. Genomes with identical hotspot profiles are represented as single branches in the dendrogram (collapsed leaves), reflecting perfect similarity in their presence–absence patterns. The presence–absence matrix was reordered to match the dendrogram leaf order. (**B**) Schematic representation of representative examples of PAPI-1-encoded GI-Hotspots 1, 2, and 3. Conserved boundaries are represented with a coloured outline and defence systems, anti-defence proteins and other conflict systems are represented in coloured arrows. (**C**) Stacked bar plot displaying the percentage of defence systems, anti-defence systems, AMR genes and other proteins (including other conflict systems, uncharacterized proteins and core ICE proteins) found within GI-Hotspots 1, 2, and 3. (**D**) Barplot showing the prevalence of defence and anti-defence systems found in GI-Hotspot 1. (**E**) Barplot showing the prevalence of defence and anti-defence systems found in GI-Hotspot 2. (**F**) Barplot showing the prevalence of defence and anti-defence systems found in GI-Hotspot 3. For panels (D–F), bar plots show the number of occurrences of each defence or anti-defence system within individual hotspots across PAPI-1 islands. The total number of hotspot instances (*n*) is indicated above each bar chart. GI-Hotspots were identified from a dataset of 1849 PAPI-1 islands (BLASTn-derived). Dataset sizes differ, so absolute proportions should not be directly compared across hotspots. Anti-defence systems are depicted in bright orange and defence systems in pale orange.

Using this approach, we identified three distinct hotspots within the PAPI-1/PAGI-5 islands: GI-Hotspot 1, defined by DUF3275 and DUF2859; GI-Hotspot 2, defined by TraG and a putative helicase; and GI-Hotspot 3, defined by TraU and TraC (Fig. 3A and B). The clustering of defence systems near TraG, TraU, and TraC suggests a potential enrichment of defence mechanisms within conjugation-associated regions of PAPI-1/PAGI-5. While it is well established that defence systems are frequently transferred via HGT and encoded on ICEs, their specific co-localization with conjugation-associated genes raises the possibility that these loci serve as preferred integration sites for defence elements. Given the selective advantage conferred by defence systems, this organization may facilitate the coordinated transfer of both conjugation machinery and defence systems, potentially enhancing the stability and persistence of ICEs in a new host [35, 44]. Our analysis further revealed that in ∼94% of cases, at least two of these hotspots co-exist on PAPI-1/PAGI-5, with the GI-Hotspot 1–GI-Hotspot 2 pair being the most frequent (∼66% of cases). In the remaining instances, all three hotspots are present (Fig. 3A).

To fully characterize the repertoire of genes encoded within each hotspot, we used the PFAM database, PADLOC, DefenseFinder, and AMRfinder [2, 45–47]. This approach revealed that GI-Hotspot 1 encodes either a defence or anti-defence system in 34% of instances, with a notable enrichment of anti-CRISPR proteins (Fig. 3B–D and Supplementary Tables S8–S11). A minority of GI-Hotspot 1 instances also encode antimicrobial resistance (AMR) genes (Fig. 3B and C, and Supplementary Table S10). In contrast, GI-Hotspots 2 and 3 carry defence systems in 21% and 13% of cases, respectively (Fig. 3C–F, and Supplementary Tables S8 and S9). Additionally, Hotspot 2 harbours a high abundance of known Type II toxin–antitoxin (TA) systems, present in 13.2% cases (Fig. 3E and Supplementary Table S11). These could either serve as additional defence strategies or as addiction modules to promote the maintenance of PAPI-1/PAGI-5 in the host. GI-Hotspot 2 stands out as the region with the highest diversity of defence systems, with Gabija and Avast IV being the most abundant (Fig. 3E). Conversely, GI-Hotspot 3 diversity is more limited, encoding only RM Type II and PD-λ-5 systems (Fig. 3F). Beyond these, each hotspot encodes transposases, integrases, phage-like proteins, and numerous proteins that remain uncharacterized following our analysis (Fig. 3C and Supplementary Table S11). In rare cases, in GI-Hotspot 2, we also identified a predicted cytolethal distending toxin A/C domain (*n* = 2) and a homologue of the epsilon toxin (ETX) (*n* = 1) (Supplementary Table S11).

Next, for genomes carrying PAPI-1, we compared the distribution of defence or anti-defence systems located within GI-Hotspots 1–3 to those found elsewhere in the same genomes. GI-Hotspot 1 is a reservoir for CRISPR–Cas I-C, almost entirely capturing this CRISPR–Cas subtype, whereas GI-Hotspot 3 showed a similarly marked enrichment for PD-λ-5 (Supplementary Fig. S2A). Other systems, including DUF4238 and CBASS, exhibited a more balanced split between GI-Hotspot 2 and elsewhere (Supplementary Fig. S2A), suggesting mobility between multiple genomic contexts. Furthermore, analysis of anti-defence systems revealed that GI-Hotspot 1 often carries specific Acr proteins, with several types occurring at higher frequencies relative to elsewhere in the genome (Supplementary Fig. S2B). Most notably, the majority of AcrIF12, AcrIE1, and AcrIF3 instances are located in GI-Hotspot 1 (Supplementary Fig. S2B).

Since our searches for homologues of the PAPI-1/PAGI-5 islands and their associated hotspots may have been constrained by the use of the BLASTn algorithm, we further investigated whether GI-Hotspots 1, 2, and 3 are present in other *Pseudomonas* and bacterial species using cblaster [48]. In cblaster, input queries (protein FASTA sequences or NCBI accessions) are submitted to BLASTp, and the resulting hits are retrieved together with their genomic context using the Identical Protein Groups (IPG) resource. Each hit can thus be mapped back to its genomic coordinates and grouped by scaffold and organism. Co-localization of genes is then defined if the hits co-occur within a user-defined maximum intergenic distance, enabling the identification of proteins that co-localize within genomes [48]. By using the boundaries of each hotspot as queries, this approach not only enabled the detection of more divergent homologues, but also allowed the identification of hotspot regions potentially residing on degenerated PAPI-1/PAGI-5 or other ICE-like elements, which may have been missed using nucleotide-level searches alone (Supplementary Fig. S1 and Supplementary Table S12). All three hotspots were predominantly identified across the *Pseudomonas* genus, with the highest enrichment observed in *P. aeruginosa* (Supplementary Fig. S3A). Notably, GI-Hotspot 1 appears to be restricted to *Pseudomonas* spp., whereas GI-Hotspots 2 and 3 were also detected in several other genera, including *Serratia, Achromobacter, Burkholderia*, and *Xanthomonas* (Supplementary Fig. S3A). As expected, in many cases these hotspots appear to be localized within genomic regions that do not share nucleotide sequence similarity with PAPI-1/PAGI-5 and are not readily predicted as canonical ICEs by tools such as ICEFinder [49] (Supplementary Table S12). This suggests that these defence hotspots may persist in remnants of degenerated or atypical ICEs (Supplementary Table S12) or may have been mobilized into novel genomic contexts through recombination or horizontal gene transfer. Furthermore, when identified in species other than *P. aeruginosa*, each hotspot encodes a defence system or anti-defence protein in at least 10% of cases (Supplementary Tables S12–S14).

Interestingly, the tendency of GI-Hotspot 1 to accumulate anti-defence proteins is consistently observed across the cblaster-derived dataset, with 25.25% of instances encoding an anti-defence protein and 10.29% encoding a defence system, encompassing a total of 17 distinct defence systems (Supplementary Fig. S3B). GI-Hotspots 2 and 3 are occupied by a known system in 17% and 5.5% of cases, respectively, across the broader cblaster dataset, and each encodes ~50 distinct systems (Supplementary Fig. S3C and D). Notably, while GI-Hotspot 3 retains its enrichment for RM Type II and PD-λ-5 systems, it exhibits greater diversity in defence systems when identified through cblaster (Supplementary Fig. S3D). Overall, these findings underscore the functional specialization and evolutionary persistence of each hotspot across diverse genomic contexts.

### Defence systems constitute the majority of PAPI-2 island length

PAPI-2 predominantly encode the exotoxin ExoU, a key virulence factor that has been used to define PAPI-2 islands in *P. aeruginosa* [32, 34], along with a high number of proteins associated with defence (Fig. 2A). Nevertheless, when we applied the same approach used for PAPI-1/PAGI-5—clustering protein pairs and using HMMs to define potential hotspots—we were unable to identify discrete defence/anti-defence hotspots within PAPI-2 islands.

**Figure 4.**
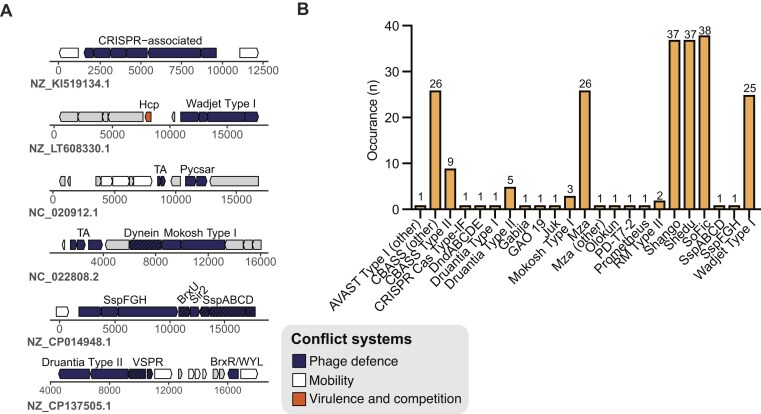
PAPI-2 islands accumulate defence systems across their whole length. (**A**) Schematic representation of representative examples of PAPI-2 islands. Defence systems, anti-defence proteins and other conflict systems are represented in coloured arrows as specified in the legend. (**B**) Barplot showing the prevalence of defence and anti-defence systems found in PAPI-2. Bar plots show the number of occurrences of each defence system within PAPI-2 islands (*n* = 868).

Instead, we found that, in most cases, defence systems are distributed throughout the majority of the PAPI-2 island length, often accompanied solely by transposases, integrases, or proteins linked to integrative conjugative elements (Fig. 4A). Within the PAPI-2 instances identified with our method, we observed that CBASS, Shedu, Shango, SoFic and Wadjet are the most abundant defence and anti-plasmid systems (Fig. 4B). Notably, PAPI-2 islands were found to lack anti-defence genes, and only a minority contained AMR genes (Fig. 4B).

In summary, our analysis shows that PAPI-2 islands are predominantly composed of defence systems distributed across their entire length, with the remainder consisting of the ExoU toxin or core GI-associated genes.

### PAGI-1 islands carry a multitude of variable hotspots

**Figure 5.**
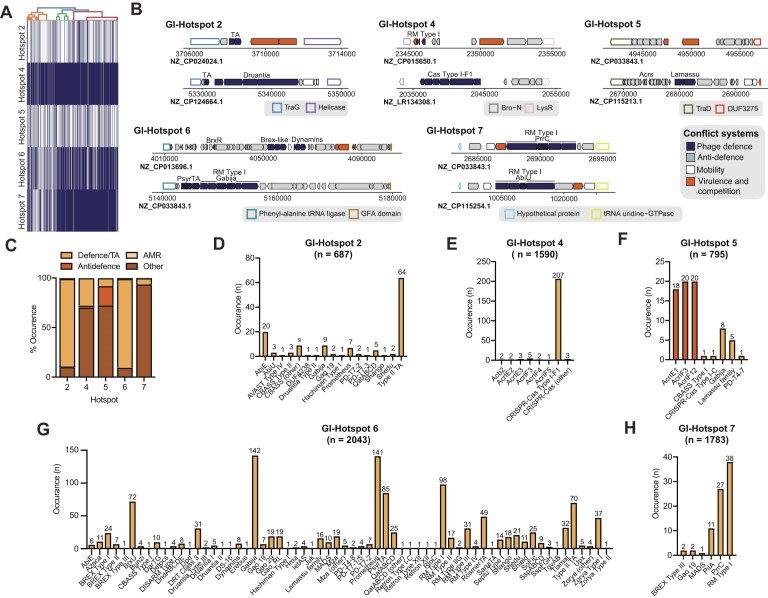
PAGI-1 carries five distinct variable hotspots. (**A**) The presence–absence matrix visualizing the distribution of the five PAGI-1 hotspots, GI-Hotspots 2, 4, 5, 6, and 7. Each row corresponds to a hotspot, while each column represents a distinct genome (nucleotide accession). Presence is indicated in dark blue and absence in white. The corresponding dendrogram groups genomes based on similarity in hotspot content, using Ward’s linkage and Euclidean distance for hierarchical clustering. Genomes with identical hotspot are collapsed together, reflecting a perfect match in their presence/absence patterns. (**B**) Schematic depiction of representative examples of PAGI-1-encoded GI-Hotspots 2, 4, 5, 6, and 7. Conserved boundaries are represented with a coloured outline and defence systems, anti-defence proteins and other conflict systems are represented in coloured arrows as specified in the legend. (**C**) Stacked bar plot displaying the percentage of defence systems, anti-defence systems, AMR genes, and other proteins (includes other conflict systems, uncharacterized proteins, and core ICE proteins) found within PAGI-1 GI-Hotspots 2, 4, 5, 6, and 7. (**D–H**) Barplot showing the prevalence of defence and anti-defence systems found in GI-Hotspot 2 (D), GI-Hotspot 4 (E), GI-Hotspot 5 (F), GI-Hotspot 6 (G), and GI-Hotspot 7 (H). For panels (D–H), bar plots show the number of occurrences of each defence or anti-defence system within GI-Hotspots 2, 4, 5, 6, and 7 across PAGI-1 islands. The total number of hotspot instances (*n*) is indicated above each bar chart. GI-Hotspots were identified from a dataset of 2149 PAGI-1 islands (BLASTn-derived). Dataset sizes differ, so absolute proportions should not be directly compared across hotspots. Anti-defence systems are depicted in bright orange and defence systems in pale orange.

Employing the HMM-based clustering method described previously, we examined the BLASTn-derived PAGI-1 islands dataset (Supplementary Tables S1 and S3) and identified five distinct hotspots, GI-Hotspots 2, 4, 5, 6, and 7 (Fig. 5A and B). GI-Hotspot 2, delimited by TraG and a helicase, is the same as that found in PAPI-1/PAGI-5 (Fig. 5B). Furthermore, GI-Hotspot 5 also exhibits a partial overlap with GI-Hotspot 1, sharing one of its boundaries (DUF3275). However, GI-Hotspot 2 and GI-Hotspot 5 are more sporadic in PAGI-1 regions compared to GI-Hotspot 4, GI-Hotspot 6, and GI-Hotspot 7, which are relatively widespread (Fig. 5A). The presence of GI-Hotspot 2 in both PAGI-1 and PAPI-1/PAGI-5, along with the partial overlap between GI-Hotspot 5 and GI-Hotspot 1, suggests that recombination, HGT, or module swapping events may have shaped the composition of these regions. Similar occurrences have been documented in other bacteria; for instance, ICEs have been shown to exchange modules and alter their integration specificity, leading to genomic diversity in *Streptococcus agalactiae* and *Helicobacter pylori* [50, 51].

The majority of PAGI-1 islands encodes at least three hotspots, with GI-Hotspots 4, 6, and 7 being the most frequent combination (66.7% cases) (Fig. 5A). Through analysis with PFAM, PADLOC, DefenseFinder, and AMRFinder, we found that GI-Hotspot 2 (88.9%) and GI-Hotspot 6 (89.6%) are primarily occupied by defence systems or toxin–antitoxin systems. A similar pattern is observed for GI-Hotspot 4 (27.7%) and GI-Hotspot 7 (10%) (Fig. 5B and C). Additionally, 2% of GI-Hotspot 4 is occupied by anti-defence proteins (Fig. 5C). Conversely, GI-Hotspot 5 encodes anti-defence proteins in 19.9% of cases and defence systems in 8% of instances (Fig. 5C).

Similarly to what was observed in PAPI-1/PAGI-5, instances of GI-Hotspot 2 found in PAGI-1 carry diverse defence systems and a high frequency of Type II TAs (Fig. 5B and D, and Supplementary Tables S14–S17). GI-Hotspot 4, 5, and 7 exhibit lower diversity, with GI-Hotspot 4 primarily harbouring CRISPR–Cas Type I-F, GI-Hotspot 5 showing a high frequency of anti-defence proteins, and GI-Hotspot 7 predominantly carrying RM Type I systems (Fig. 5B, E, F, and H). Notably, in most cases, RM Type I systems found in GI-Hotspot 7 contain a second defence system embedded within them, a trend previously reported in other studies [46, 52–54]. Furthermore, the predominance of anti-defence proteins in GI-Hotspot 5 mirrors a similar trend within the related GI-Hotspot 1, further reinforcing the possibility of recombination between them (Fig. 5C and F, and Fig. 2, and Supplementary Tables S9 and S16). Finally, GI-Hotspot 6 exhibits the highest diversity of defence systems observed across *Pseudomonas* GIs identified in this study, with >50 distinct systems detected (Fig. 5G). Among these, the most abundant systems include Gabija, Prometheus, and RM Type I (Fig. 5G and Supplementary Table S15). PFAM analysis of GI-Hotspot 6 proteins that remained uncharacterized following our analysis revealed that many of these harbour domains typically associated with defence, including AAA^+^ ATPase, nucleases, and Sir2 domains. However, they were not identified as part of any known defence system (Supplementary Tables S15–S17). This suggests that these regions may represent novel defence systems or subtypes; alternatively, they could correspond to known systems that exhibit substantial sequence divergence.

Furthermore, GI-Hotspots 2, 4, 6, and 7 occasionally harbour modules involved in inter-bacterial competition (e.g. colicins and pyocins) and toxins targeting eukaryotic cells (e.g. ShlA/B and RhuM) (Supplementary Table S17). However, these modules constitute only a minor fraction of the characterized proteins within these hotspots (Fig. 5C, and Supplementary Tables S15 and S17), in contrast to the higher proportions observed in other species such as *Serratia* and *Salmonella* [13, 16]. We note that the function of many proteins encoded in GI-Hotspots 2, 4, 6, and 7 remains unknown following our analysis with PFAM, PADLOC, DefenseFinder, and the VFDB.

In genomes carrying PAGI-1, we compared the contribution of each PAGI-1 hotspots to the overall repertoire of anti-phage systems found across the chromosome. Upx is almost exclusively encoded in GI-Hotspot 6. Bunzi, Dpd, Ssp, BREX type II, MADS, and Tiamat are equally distributed between GI-Hotspot 6 and other chromosomal locations. A similar pattern is found for CRISPR–Cas Type I-F1 in GI-Hotspot 4 and CBASS in GI-Hotspot 2 (Supplementary Fig. S4A). A similar comparison for anti-defence systems revealed that GI-Hotspots 4 and 5 are focal points for anti-CRISPR diversity in PAGI-1-positive genomes (Supplementary Fig. S4B).

Only AcrIF12 exhibits clear hotspot specificity, occurring almost exclusively in GI-Hotspot 5. By contrast, AcrIE3, AcrIE2, AcrIF5 (relative to GI-Hotspot 4), AcrIE1, and AcrIF3 (relative to GI-Hotspot 5) occur at similar frequencies inside and outside the hotspot (Supplementary Fig. S4B).

We next employed cblaster using the boundaries of GI-Hotspots 4, 5, 6, and 7 to investigate their broader distribution across bacterial genomes. This approach revealed that these hotspots are present in a wider range of genomes (Supplementary Table S18 and Supplementary Fig. S4C). The only exception was GI-Hotspot 6, which appears to be confined to the ∼2000 PAGI-1 islands detected via BLASTn (Supplementary Tables S1 and S18). GI-Hotspot 4 remains largely restricted to *P. aeruginosa* and other *Pseudomonas* species (Supplementary Fig. S4C). In contrast, GI-Hotspot 5 shows a broader host range, being also found in *Aquabacterium, Uliginosibacterium, Brenneria, Burkholderia*, and *Xanthomonas* (Supplementary Fig. S4C). GI-Hotspot 7 is primarily detected in *Pseudomonas*, though it can also be sporadically identified in other genera (Supplementary Fig. S4C). As with GI-Hotspots 1–3, many of these homologous regions are not localized within canonical ICEs or MGEs, nor do they share nucleotide sequence similarity with PAPI/PAGI/LESGI islands. This observation further supports the notion that these hotspots may persist within remnants of degenerated or atypical mobile elements, or may have been mobilized into novel genomic contexts [53].

Functional characterization of the cblaster-derived datasets revealed patterns consistent with our previous findings, while also expanding the diversity of defence systems associated with GI-Hotspots 4, 5 and 7. GI-Hotspot 4 was still found to predominantly encode a CRISPR–Cas Type I-F1 system (Supplementary Fig. S4D, and Supplementary Tables S19 and S20). GI-Hotspot 5 retains its strong enrichment for anti-defence mechanisms, frequently encoding AcrIE1, AcrIF12, and AcrIF3, alongside a notable presence of the Gabija system. Interestingly, compared to the BLASTn-derived dataset, the repertoire of defence systems in this hotspot is increased (Supplementary Fig. S4E). Similarly, GI-Hotspot 7 continues to show an accumulation of RM Type I and PrrC systems, often with PrrC embedded within RM loci. However, the expanded dataset also reveals a greater diversity of additional systems encoded at a lower frequency, with PifA being the most enriched amongst them (Supplementary Fig. S4F, and Supplementary Tables S19 and S20).

Together, these results underscore the modularity and evolutionary plasticity of PAGI-1-associated defence hotspots. The expanded host range and defence gene diversity uncovered through cblaster reinforces the view that these hotspots act as dynamic platforms for the dissemination of defence and anti-defence systems. Moreover, the overlap between PAGI-1 and PAPI-1, such as the shared GI-Hotspot 2 and the partial similarity between GI-Hotspots 1 and 5, highlights the potential role of horizontal gene transfer, recombination, and module exchange in shaping these regions [37, 55].

### PAGI-2, PAGI-3, and LESGI-3 share GI-Hotspot 8

**Figure 6.**
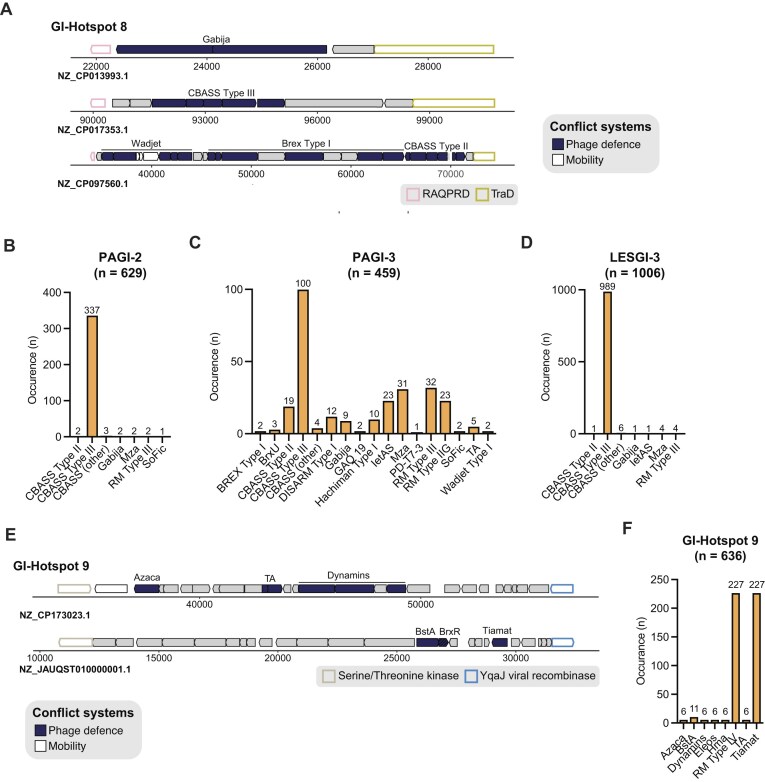
Genomic organization and defence-system abundance of GI-Hotspots 8 and 9 across *Pseudomonas* pathogenicity islands. (**A**) Schematic representation of representative examples of GI-Hotspot 8 encoded on PAGI-2, PAGI-3, and LESGI-3. Conserved boundaries are represented with a coloured outline and defence systems, anti-defence proteins, and other conflict systems are represented in coloured arrows as specified in the legend. (**B–D**) Barplot showing the prevalence of defence and anti-defence systems found in GI-Hotspot 8 encoded on PAGI-2 (B), PAGI-3 (C), and LESGI-3 (D). (**E**) Schematic representation of representative examples of GI-Hotspot 9. Boundaries are showed in coloured outline. Defence systems and anti-defence proteins are represented in coloured arrows as specified in the legend. (**F**) Barplot showing the prevalence of defence and anti-defence systems found in GI-Hotspot 9 when identified by BLASTn. For panels (B–D) and (F), bar plots show the number of occurrences of each defence system within GI-Hotspot 8 (across PAGI-2, PAGI-3, and LESGI-3) and GI-Hotspot 9 (across LESGI-1 islands). The total number of hotspot instances (*n*) is indicated above each bar chart. GI-Hotspots were identified from datasets of 629 PAGI-2, 459 PAGI-3, 1363 LESGI-3, and 636 LESGI-1 islands (BLASTn-derived), respectively. Dataset sizes differ, so absolute proportions should not be directly compared across hotspots. Anti-defence systems are depicted in bright orange and defence systems in pale orange.

PAGI-2, PAGI-3, and LESGI-3 harbour a variable region identified as GI-Hotspot 8. This region is flanked by *traD* and a RAQPRD family integrative conjugative element protein (Fig. 6A–D).

While the structural boundaries of GI-Hotspot 8 are conserved, the defence gene content slightly varies between the three islands groups (Fig. 6B–D and Supplementary Table S21). In all cases, CBASS Type III is the most frequently encoded system; however, the overall repertoire of defence systems differs between islands, with the most pronounced divergence observed in PAGI-3. Specifically, PAGI-3 encodes 17 distinct systems, in contrast to the more limited sets found in PAGI-2 and LESGI-3 (Fig. 6B–D and Supplementary Table S21).

Furthermore, no anti-defence proteins, AMR, virulence, or anti-bacterial modules were identified within GI-Hotspot 8 (Supplementary Table S22). However, consistent with other hotspots described in this study, numerous proteins within GI-Hotspot 8 harbour domains typically associated with defence against MGEs; yet are not recognized as components of known systems. A substantial proportion of proteins also remain functionally uncharacterized following analysis with PFAM and VFDB (Supplementary Tables S5 and S22).

In genomes carrying PAGI-2, PAGI-3, or LESGI-3, GI-Hotspot 8 is heavily enriched for CBASS systems compared to the remainder of the genome. Multiple CBASS subtypes dominate the hotspot, whereas the broader genome of these strains contained a more diverse mix of defence systems with CBASS at much lower representation. Other systems in GI-Hotspot 8, including restriction–modification (Types I–III), Mza, IetAS, Gabija, BrxU, SoFic, PD-T4-6, and Druantia III, occur at much lower frequencies (Supplementary Fig. S5A–C).

To assess the broader distribution of GI-Hotspot 8 beyond PAGI-2, PAGI-3, and LESGI-3 contexts, we used cblaster with its flanking genes as the query. This approach recovered over 40, 000 hits, revealing that GI-Hotspot 8 is predominantly associated with *P. aeruginosa* genomes (Supplementary Table S23 and Supplementary Fig. S6A). While the majority of retrieved regions were from *P. aeruginosa*, we also identified a smaller number of GI-Hotspot 8 instances—typically ranging from 100 to 1000 occurrences—in other *Pseudomonas* species and distantly related genera, including *Serratia, Salmonella, Klebsiella*, and *Xanthomonas*. These findings suggest that, although less frequent, GI-Hotspot 8 has disseminated across species boundaries (Supplementary Table S23 and Supplementary Fig. S6A).

Notably, 29.3% of the regions within the cblaster-derived dataset encode at least one known defence system, and CBASS Type III remains the most prevalent (Supplementary Table S24 and Supplementary Fig. S6B), consistent with previous literature [35]. Across the expanded cblaster-derived dataset, GI-Hotspot 8 was found to encode a larger and more diverse repertoire of 60 distinct systems, including more frequently-occurring systems such as RM Type III (*n* = 1542), ietAS (*n* = 797), Gabija (*n* = 527), Mza (*n* = 1338), and Lamassu systems (*n* = 475) (Supplementary Fig. S6B).

### LESGI-1 encodes GI-Hotspot 9

Through BLASTn searches, ~300 LESGI-1-like islands were identified (Supplementary Table S1). LESGI-1 islands encode GI-Hotspot 9, flanked by a serine/threonine kinase and a YqaJ-like recombinase (Fig. 6E). The majority of GI-Hotspot 9 instances encode Tiamat, RM Type IV, BstA, Hma, Azaca, Type II TA, or Dynamins, with Tiamat and RM Type IV being the most abundant (Fig. 6E and F, and Supplementary Table S25). Additionally, GI-Hotspot 9 frequently carries phage-like proteins. These are predicted to encode structural proteins, such as capsid and head proteins, thus suggesting they likely represent remnants of prophage ancestry rather than indicators of current mobility of LESGI-1 (Fig. 6E and F, and Supplementary Table S26).

In genomes carrying LESGI-1, GI-Hotspot 9 is dominated by a narrow set of defence systems that appear proportionally more often in the hotspot than in the rest of the genome. Tiamat, RM Type IV, and BstA are the most frequent, with Tiamat and RM Type IV present in over three-quarters of GI-Hotspot 9 instances and BstA in more than half, compared to far lower representation elsewhere in the same genomes (Supplementary Fig. S7A). Dynamins, Azaca, and Hma are also often present in the hotspot, although at lower absolute counts (Supplementary Fig. S7A).

The cblaster analysis detected GI-Hotspot 9 in ~1000 genomes, the vast majority of which belong to *Pseudomonas* species, with a small number found in *Aeromonas, Burkholderia*, and *Acinetobacter* (*n* = 15 non-*Pseudomonas* genomes in total) [26]. Across this expanded dataset, Tiamat and RM Type IV remain the most frequently encoded defence system (Supplementary Fig. S7B and Supplementary Table S28). Although no complete defence systems are predicted in the few non-*Pseudomonas* representatives, PFAM analysis identified genes encoding domains commonly associated with defence against MGEs, such as N-6 DNA methylases, HerA helicase, and RM-like elements, suggesting the presence of novel defence system, new subtypes, or known systems with significant sequence divergence (Supplementary Table S29).

### LESGI-11 carries two hotspots

**Figure 7.**
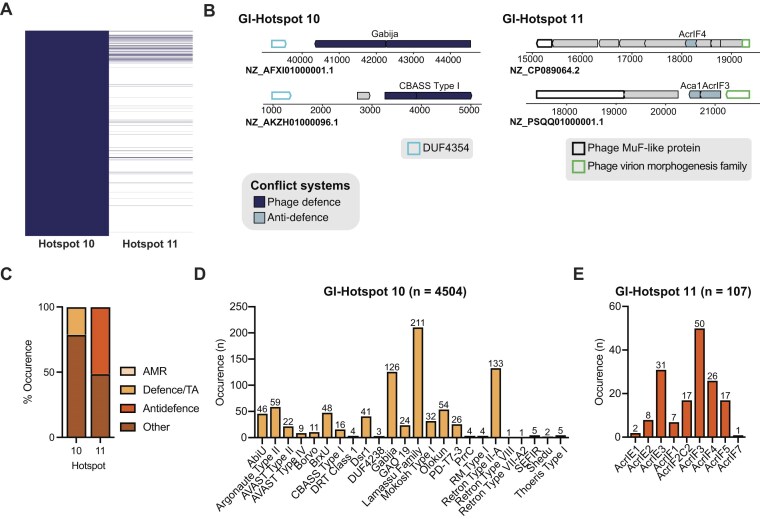
LESGI-11 Hotspots, GI-Hotspots 10 and 11, are specialized for defence or anti-defence cargo, respectively. (**A**) The presence–absence matrix visualizing the distribution of the two LESGI-11 hotspots: GI-Hotspots 10 and 11. Each row corresponds to a hotspot, while each column represents a distinct genome (nucleotide accession). Presence is indicated in dark blue and absence in white. (**B**) Schematic representation of representative examples of GI-Hotspots 10 and 11. Conserved boundaries are represented with a coloured outline and defence systems, and anti-defence proteins are represented in coloured arrows as specified in the legend. (**C**) Stacked bar plot displaying the percentage of defence systems, anti-defence systems, AMR genes, and other proteins (includes other conflict systems, uncharacterized proteins, and core ICE proteins) found within each of GI-Hotspots 10 and 11. (**D** and **E**) Barplot showing the prevalence of defence and anti-defence systems found in GI-Hotspot 10 (**D**) and GI-Hotspot 11 (**E**). For panels (D and E), bar plots show the number of occurrences of each defence or anti-defence system within individual hotspots across LESGI-11 islands. The total number of hotspot instances (*n*) is indicated above each bar chart. GI-Hotspots 10 and 11 were identified from a dataset of 4 504 LESGI-11 islands (BLASTn-derived). Dataset sizes differ, so absolute proportions should not be directly compared across hotspots. Anti-defence systems are depicted in bright orange and defence systems in pale orange.

Our HMM-based clustering method applied to a BLASTn-derived dataset of LESGI-11 islands identified two additional hotspots: GI-Hotspots 10 and 11. GI-Hotspot 11, which is found in only ∼6% of LESGI-11 islands (Fig. 7A and B), is consistently flanked by genes encoding a phage Mu protein F and a phage virion morphogenesis family protein. In contrast, GI-Hotspot 10 is present in all LESGI-11 regions and located at the terminal end of each island, delimited on one side by a DUF4354 domain-containing gene. The opposite end lacks a conserved boundary and is instead fully occupied by defence systems (Fig. 7A and B).

GI-Hotspot 10 harbours defence systems in ∼20% of cases, whereas GI-Hotspot 11 encodes an anti-defence protein in ~50% of instances (Fig. 7C, and Supplementary Tables S30 and S31). Beyond the identified defensive elements, both hotspots largely consist of uncharacterized proteins, structural genes, and phage-like components (Supplementary Table S32), with no evidence of additional conflict systems such as virulence or interbacterial antagonism modules, or AMR genes (Supplementary Tables S30–S32). GI-Hotspot 10 encodes 24 distinct defence systems, with Gabija, Lamassu, and Retron IIA being most prevalent (Fig. 7D). The anti-defence repertoire of GI-Hotspot 11 is instead predominantly composed of anti-CRISPR proteins (Fig. 7E).

Retron II-A, Olokun, Argonaute I, and AVAST II and IV are almost exclusively found on GI-Hostpot 10 (each present in ≥70%–90% of GI-Hotspot 10 instances) compared to elsewhere in the genome (Supplementary Fig. S8A). Additional systems such as AbiU, PD-T7-3, Borvo, Lamassu, SEFIR, and BrxU also occur often within GI-Hotspot 10, although their distribution is more balanced, with roughly equal representation between the hotspot and elsewhere in the genome (Supplementary Fig. S8A). GI-Hotspot 11 is dominated by anti-CRISPR genes, several of which are markedly enriched relative to the genomic background (Supplementary Fig. S8B). The most frequent are AcrIE3, AcrIF2 c2, AcrIF1, AcrIE2, and AcrIF5, with AcrIE3 and AcrIF2 c2 approaching saturation within the hotspot (Supplementary Fig. S8B).

This functional segregation—with GI-Hotspot 10 encoding exclusively defence systems and GI-Hotspot 11 carrying only anti-defence proteins—was preserved in an expanded dataset obtained through cblaster (Supplementary Fig. S8C). GI-Hotspot 10 was identified in ~21, 000 genomes, all belonging to *P. aeruginosa*, while GI-Hotspot 11 was detected in ∼7000 genomes, primarily *Pseudomonas*, with a minority of hits in *A. baumannii* and *Yersinia enterocolitica* (Supplementary Fig. S8C and Supplementary Table S33). Notably, 23.4% of GI-Hotspot 10 instances and 71% of GI-Hotspot 11 instances identified through cblaster encoded a known defence or anti-defence system, respectively (Supplementary Tables S33–S36). As observed for other hotspots identified by cblaster, GI-Hotspot 10 and 11 regions are not consistently embedded within canonical LESGI-like islands or ICEs, suggesting that these hotspots can be acquired independently of typical island boundaries (Supplementary Table S33).

Across the expanded dataset, GI-Hotspot 10 encompasses 30 distinct defence systems, with Gabija, Lamassu, and Retron IIA remaining among the most prevalent, alongside additional enrichment for Thoeris I, Argonaute I, and Borvo (Supplementary Table S34 and Supplementary Fig. S8D). Conversely, GI-Hotspot 11 remains enriched in anti-CRISPR proteins (Supplementary Table S35 and Supplementary Fig. S8E). Together, these results demonstrate the distinct yet complementary roles of GI-Hotspots 10 and 11 in encoding defence and anti-defence systems on LESGI-11, respectively.

### Statistical validation of GI-Hotspots

To confirm that GI-Hotspots 1–11 represent genuine sites of defence and anti-defence system enrichment, rather than random occurrences, we evaluated the frequency of system occupancy at each hotspot across the entire correspondent dataset obtained through cblaster. We assumed a background probability of 5% for random insertion events. For each hotspot, we calculated the proportion of genomes containing a defence or anti-defence system and performed a one-sided binomial test to determine whether this proportion was significantly greater than the expected background frequency. Additionally, we integrated a Bayesian analysis by modelling the occupancy proportion with a Beta posterior distribution (using a uniform prior) to estimate the probability that the true occupancy proportion exceeds the 5% background level.

The analysis revealed that all identified hotspots show a significantly higher frequency of defence and anti-defence system occupancy than expected by chance (Table 2). Observed proportions of genomes containing defence or anti-defence systems at these hotspots ranged from 5.5% (Hotspot 3) to 98.4% (Hotspot 9), with fold enrichments spanning 1.10× to 19.68× above the background. All hotspots exhibited highly significant one-sided binomial test *P*-values (all *P* < 1 × 10⁻⁵), supporting strong enrichment (Table 2).

**Table 2.** Statistical enrichment of defence systems across GI-Hotspots 1–11

Hotspot	Genomes with system	Total genomes	Proportion (%)	Fold enrichment	*P*-value	Bonferroni-corrected *P*-value	Bayesian *P* (*P* > 0.05) (%)
Hotspot 1	2878	11 189	25.72	5.14×	<1e-10	<1.1e-9	100.00
Hotspot 2	6774	39 588	17.11	3.42×	<1e-10	<1.1e-9	100.00
Hotspot 3	2153	39 181	5.50	1.10×	4.92e-06	5.41e-5	99.9996
Hotspot 4	5170	17 407	29.70	5.94×	<1e-10	<1.1e-9	100.00
Hotspot 5	5820	29 249	19.90	3.98×	<1e-10	<1.1e-9	100.00
Hotspot 6	3499	3905	89.60	17.92×	<1e-10	<1.1e-9	100.00
Hotspot 7	2505	25 050	10.00	2.00×	≈0	≈0	100.00
Hotspot 8	12 409	41 913	29.61	5.92×	<1e-10	<1.1e-9	100.00
Hotspot 9	1060	1077	98.42	19.68×	<1e-10	<1.1e-9	100.00
Hotspot 10	4853	21 091	23.01	4.60×	<1e-10	<1.1e-9	100.00
Hotspot 11	5215	7765	67.16	13.43×	<1e-10	<1.1e-9	100.00

The Bayesian analysis further confirmed these findings, with posterior probabilities that the true occupancy proportion exceeds 5% (Table 2). Notably, GI-Hotspots 6, 9, and 11 displayed the highest occupancy proportions (89.6%, 98.4%, and 67.2%, respectively) and the greatest fold enrichments (up to ∼20×), highlighting them as especially prominent loci for defence system integration (Table 2).

Taken together, our results demonstrate that these hotspots are significantly enriched for defence and anti-defence systems beyond random occurrences, confirming their status as *bona fide* variable defence hotspots.

## Discussion

It is well established that MGEs, including plasmids, prophages and integrative and conjugative elements (ICEs), frequently accumulate defence and anti-defence systems, often in discrete hotspots, to enhance their own stability and facilitate host survival under phage attack [9, 10, 14, 18, 26, 27, 56].

A recent large-scale study of over 13, 000 chromosomally integrated MGEs (ciMGEs) confirmed that defence, AMR, and virulence genes are widespread across diverse bacterial and archaeal phyla, with ICEs emerging as key hotspots for their accumulation [57]. More recently, Armán *et al.* reported the presence of Acr proteins within newly described PAGI-2-like elements in clinical *P. aeruginosa* isolates, indicating that MGEs also act as reservoirs of anti-defence genes [26]. However, these studies offered broad cargo surveys of ciMGEs and novel PAGI islands, without delineating boundary-defined hotspots or comparing repertoire differences in defence/anti-defence systems content across hotspots and island families.

In *P. aeruginosa*, PAPI-1 and PAGI-5 represent well-characterized integrative conjugative elements, capable of conjugative transfer and known for encoding virulence factors [30–32]. Furthermore, more recent evidence highlighted that PAGI-2, PAGI-3, and LESGI-3 are ICE-like elements that show partial synteny to known ICEs [35]. These islands carry some genes related to conjugative transfer; however, unlike PAPI-1 and PAGI-5, their ability to self-transfer has not been experimentally demonstrated [30, 35]. In contrast, PAPI-2 and most PAGI-X and LESGI-X islands do not conform to the classical ICE architecture and instead represent degenerate or atypical genomic islands lacking conjugation modules [29, 30, 32, 35]. The extent to which PAPI-X, PAGI-X, and LESGI-X contribute to the distribution and organization of defence systems in *P. aeruginosa* has not been systematically examined.

Our study directly addresses these knowledge gaps by mapping, delimiting, and analysing the local organization of defence and anti-defence systems in *P. aeruginosa* PAPI-X, PAGI-X, and LESGI-X islands. Using a combination of BLASTn, protein-level homology via cblaster, and HMM-guided hotspot prediction, we uncover eleven discrete hotspots encoded within *P. aeruginosa* genomic islands of the PAPI-X, PAGI-X, or LESGI-X families. This represents a substantial expansion of the known repertoire of MGE- and genomic islands-associated defence hotspots in this species. Prior to this work, only two hotspots—cDHS1 and cDHS2—had been described [38]; however, these loci are not frequently associated with known ICEs or with the PAPI/PAGI/LESGI island families [38]. Beyond this expansion, we also show that PAPI-2 exhibits a whole-island defence-enriched architecture rather than discrete hotspots, and that hotspots shared across distinct island families harbour divergent repertoires of defence and anti-defence systems. We note that our delimitation of defence and anti-defence-enriched hotspots is based on computational inference and has not yet been functionally tested. Future work using targeted deletions or heterologous expression will be required to establish their direct contribution to resistance against phages and other MGEs, and to clarify how these loci operate within the broader defence repertoire in which they are embedded.

Our study further reports that the 11 identified hotspots are well-defined and spatially constrained in PAPI-1, PAGI-1, PAGI-2, PAGI-3, LESGI-1, LESGI-3, and LESGI-11. This suggests that genomic modularity—i.e. the clustering of defence genes into discrete loci—is maintained independently of conjugative potential, highlighting that PAPI-X/PAGI-X/LESGI-X genomic islands (not just ICEs) can contribute to the organization of bacterial immunity.

Beyond their structural modularity, hotspots also display strong functional biases when compared against the broader chromosomal background. Individual hotspots concentrate specific immune systems (CBASS subtypes, Upx, Tiamat, Olokun, Retron II-A, etc.) or anti-defence proteins (mainly anti-CRISPR proteins) (Supplementary Figs S2, S4A, S5, S7A, and S8A and B). This pattern suggests that hotspots not only provide structural clustering but also serve as preferential repositories, enriching for certain defence or anti-defence functions far beyond their genomic baseline.

Notably, GI-Hotspots 1, 2, and 5, in PAPI-1/PAGI-5 and PAGI-1, respectively, are flanked by conjugation genes (*traG, traU, traC*, and *traD*). The co-localization of defence and conjugation loci suggests that some GIs may be structurally organized to promote the coordinated mobilization of both transfer and immunity roles. Functionally, this could ensure that upon entering a new host, the mobilized island—whether a self-mobilizing ICE or a GI transferred through another HGT mechanism—provides immediate defence protection, safeguarding both the host and the element itself during transfer and optimizing the success and persistence of these elements [58, 59]. Similar patterns have been observed in other species. For instance, the ICEBs1 element in *Bacillus subtilis* encodes the defence gene *spbK*, which prevents infection by phage SPβ and thereby protects the host population. Importantly, this element also retains a fully functional conjugation system. Another example is CampyICE1 in *Campylobacter jejuni* and *Campylobacter coli*. CampyICE1 encodes a degenerate Type II-C CRISPR–Cas with a functional Cas9. CampyICE1 arrays target conjugative plasmids, leading to exclusion from the host [60, 61].

Notably, several hotspots also encode putative anti-defence systems, which are expected to interfere with host-encoded immunity (Figs 2–5). This is consistent with recently findings, highlighting the presence of anti-CRISPR proteins in novel PAGI-2-like islands in clinical *P. aeruginosa* isolates [26]. While such proteins may weaken host resistance to lytic phages and other incoming MGEs, they could confer protection to the PAPI-X/PAGI-X/LESGI-X island during transfer, particularly from host-encoded immunity that could otherwise restrict its establishment. In this context, the co-occurrence of anti-defence and defence systems within the same island in separate hotspots, as well as the presence of multiple PAPI-X, PAGI-X, and LESGI-X combinations in individual strains, may reflect a functional arrangement: anti-defence genes enable the island to bypass host immunity, while the co-localized, diverse defence systems provide broader protection against other incoming MGEs, helping to stabilize the host–MGE association post-transfer. For instance, the Type I-C CRISPR–Cas encoded on some PAPI-1 islands is able to prevent infection from several lytic phages, providing post-integration immunity [58]. Nevertheless, other phages and MGEs encode multiple anti–CRISPR proteins that are able to counter the PAPI-1-encoded Type I-C CRISPR–Cas, illustrating the complexity of phage-host-MGEs arms race and co-existence [58]. Similar strategies are observed in conjugative plasmids, where anti-CRISPR and anti-restriction genes are positioned in the leading region and expressed during transfer to block recipient defences [27, 62].

GI-Hotspots 1–11 modularity is not merely structural, but functional. Although the broader genomic islands in which these hotspots are embedded often carry diverse functions—including virulence factors, anti-bacterial toxins, or secondary metabolite synthesis— the defence hotspots themselves are composed preferentially of defence or anti-defence genes. Among the genes we were able to annotate, we did not detect any significant enrichment of other conflict modules. However, several hotspots also contain numerous genes that we were unable to characterize with our current methods, suggesting the presence of unannotated or novel functions. This compartmentalized architecture contrasts with that observed in other bacteria such as *Serratia, Vibrio, Salmonella, E. coli*, and several Enterobacteria, where conflict modules—including anti-bacterial toxins, defence systems, and virulence factors—often co-localize within shared genomic boundaries [13, 15, 16], pointing to genus-specific strategies in the organization of defensive and offensive modules.

In addition, several hotspots are conserved across island families. GI-Hotspot 2 (PAGI-1 versus PAPI-1), GI-Hotspot 8 (PAGI-2/3 versus LESGI-3), and the GI-Hotspot 1/5 partial boundary overlap each show that the same hotspot carries island-/family-specific repertoires, although some systems are shared. A parallel pattern is observed in *Serratia* Island 3, which is widespread across several *Enterobacteriaceae* species and consistently retains its boundaries, while carrying highly divergent cargos of defence systems, virulence factors, and interbacterial competition modules [13].

Whilst we identified 11 demarcated hotspots in several PAPI-X, PAGI-X, and LESGI-X islands, PAPI-2 shows a different trend, accumulating numerous defence systems across its entire length (Fig. 4) [13, 15, 16]. Comparable whole-island architectures occur in *Serratia* defence islands, *Salmonella enterica* ST313 islands, and the seventh-pandemic islands VSP-I/II and VPI-2 of *Vibrio cholerae*, each of which carries multiple distinct defence modules distributed across their sequences [53, 54, 63]. These parallels indicate that whole-island defence loading, as observed for PAPI-2, represents a recurring organizational strategy in diverse bacteria. Furthermore, PAPI-2 islands also carry the virulence factor ExoU, suggesting that these elements act as multi-conflict islands, dedicated to defence against MGEs and host invasion, similarly to what is seen in other species such as *Serratia, Salmonella* and *Vibrio* [13, 15, 16].

Notably, not all PAPI-X, PAGI-X, or LESGI-X islands contained identifiable defence systems. Several, including PAGI-9, PAGI-10, PAGI-11, and certain LESGI-X islands, were instead dominated by genes of unknown function (Fig. 1). As shown in previous studies, these GIs can carry modules for secretion, metabolism, and secondary-metabolite biosynthesis. For example, PAGI-9 and PAGI-10 are Rhs-like elements lacking VgrG/Hcp; LESGI-8 encodes Type II secretion components; LESGI-9 carries partial clusters (NADH dehydrogenases, methyltransferases, uncharacterized proteins, and a secreted protein); LESGI-12 includes an AMR-associated porin; LESGI-16 encodes multiple enzymes and tRNAs; and LESGI-2 harbours a pyoluteorin biosynthetic cluster [29, 43]. These uncharacterized regions may encode novel defence systems, secondary metabolite pathways, virulence, or anti-bacterial effectors. Experimental investigation or the use of AI-based prediction tools such as DefensePredictor may aid to elucidate their biological roles [64, 65].

Interestingly, the 11 identified hotspots also show non-random internal composition: some are predominantly defence-associated, others are enriched for anti-defence functions, and a few are biased towards a particular system, such as CRISPR–Cas type I-F1 in GI-Hotspot 4 or RM systems in GI-Hotspot 7, alongside a sparse mix of others. This pattern may be consistent with selective pressures under specific ecological conditions, as previously noted for hotspots carrying only abortive infection systems in *Acinetobacter*, Thoeris Type I in *Serratia* defence island 1 or anti-defence-dominated regions on conjugative plasmids [13, 27, 66] Analogous tendencies to preferentially accumulate either defence or anti-defence modules have also been observed in the previously identified cDHS1 and cDHS2 hotspots in *P. aeruginosa*, which exclusively encode defence systems [38].

Taken together, we delineate 11 defence hotspots in *P. aeruginosa* that are enriched for defence or anti-defence systems, show that conserved hotspots harbour divergent repertoires across island families, and identify a whole-island defence-enriched architecture in PAPI-2. Collectively, these findings refine how PAPI-, PAGI-, and LESGI-family islands shape the species’ immunity repertoire and suggest that co-encoded anti-defence systems may contribute to island persistence by counteracting chromosomally encoded defences.

## Supplementary Material

lqaf148_Supplemental_Files

## Data Availability

All the data generated in this study and necessary for interpretation are provided in the Main and Supplementary Information. Custom scripts utilized for the analysis are deposited at https://github.com/GM110Z/PAPI-islands-analysis and https://doi.org/10.5281/zenodo.17244225.
