# Supplementary material for: Genomic islands in Pseudomonas encode modular hotspots of defence and anti-defence systems
Source: NAR Genom Bioinform. 2025 Nov 19;7(4):lqaf148. doi: 10.1093/nargab/lqaf148 (PMC12629846; doi:10.1093/nargab/lqaf148)
Supplement: lqaf148_Supplemental_Files [file lqaf148_supplemental_files.zip › Supplementary.pdf]

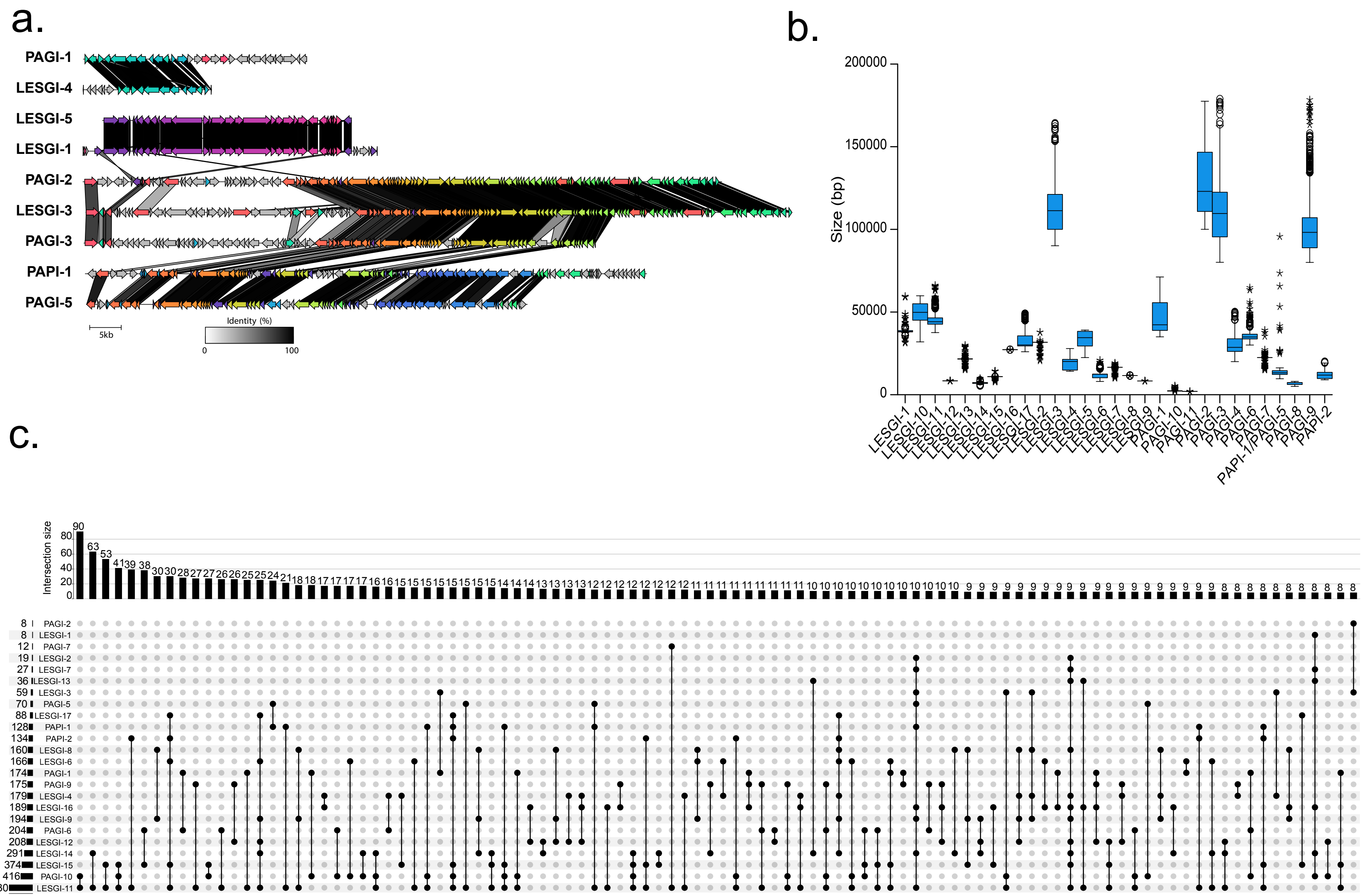
a.
PAGI-1
LESGI-4
LESGI-5
LESGI-1
PAGI-2
LESGI-3
PAGI-3
PAPI-1
PAGI-5
5kb
Identity (%)
0
100
b.
Size (bp)
200000
150000
100000
50000
0
LESGI-1
LESGI-10
LESGI-11
LESGI-12
LESGI-13
LESGI-14
LESGI-15
LESGI-16
LESGI-17
LESGI-2
LESGI-3
LESGI-4
LESGI-5
LESGI-6
LESGI-7
LESGI-8
LESGI-9
PAGI-1
PAGI-10
PAGI-11
PAGI-2
PAGI-3
PAGI-4
PAGI-6
PAGI-7
PAPI-1/PAGI-5
PAGI-8
PAGI-9
PAPI-2
c.
Intersection size
8 PAGI-2
8 LESGI-1
12 PAGI-7
19 LESGI-2
27 LESGI-7
36 LESGI-13
59 LESGI-3
70 PAGI-5
88 LESGI-17
128 PAPI-1
134 PAPI-2
160 LESGI-8
166 LESGI-6
174 PAGI-1
175 PAGI-9
179 LESGI-4
189 LESGI-16
194 LESGI-9
204 PAGI-6
208 LESGI-12
291 LESGI-14
374 LESGI-15
416 PAGI-10
830 LESGI-11

Supplementary Figure 1. *Pseudomonas* genomic islands of the PAPI-X, PAGI-X and LESGI-X family have a variable size. (a) Clinker nucleotide alignment of representative sequences for each PAGI-X, PAPI-X and LESGI-X group. Homologous genes are shaded in the same colours, and the black/grey blocks indicate level of identity across sequences. **(b)** Boxplot showing the average length (bp) of PAPI-X, PAGI-X and LESGI-X islands, identified through BLASTn searches. **(c)** UpSet plot of the 100 most frequent GI combinations across assemblies, excluding single-island occurrences. The bottom matrix indicates which genomic islands are present in each combination: filled dots represent inclusion of an island in that set, and connected dots indicate multi-island combinations. The vertical bars above each column show how many assemblies (NCBI accessions) contain that specific combination of islands. The horizontal bars on the left indicate the total number of genomes in which each individual island appears, regardless of combination. Only the top 100 most frequent multi-island combinations (by count) are displayed. The complete dataset including ~4900 combination and their frequency of occurrence (by count) is shown in **Supplementary Table 4.**

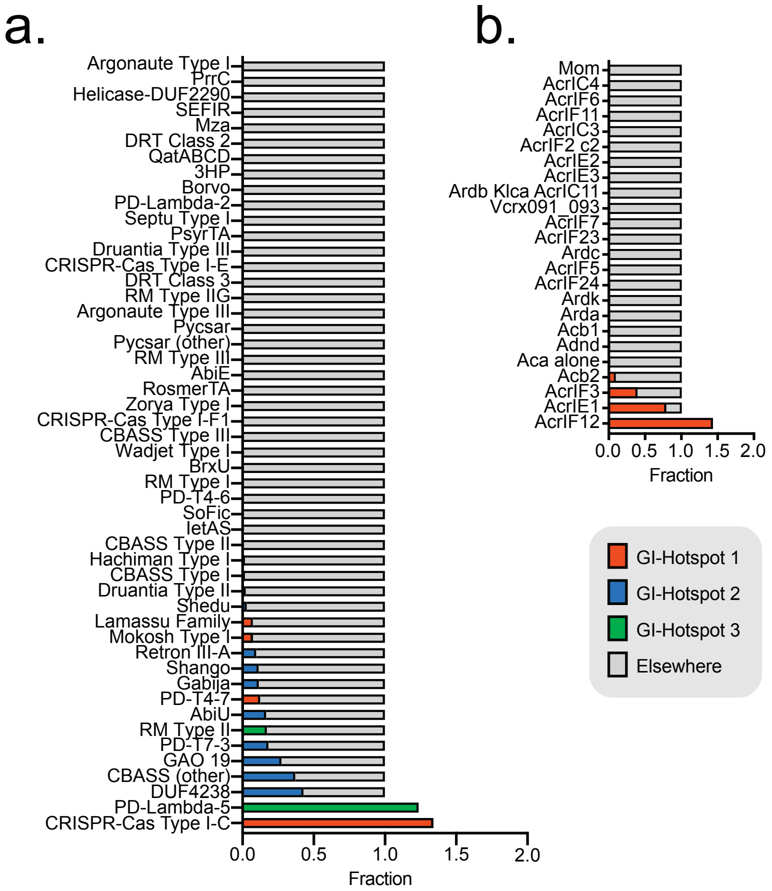

a.
Argonaute Type I
PrrC
Helicase-DUF2290
SEFIR
Mza
DRT Class 2
QatABCD
3HP
Borvo
PD-Lambda-2
Septu Type I
PsyrTA
Druantia Type III
CRISPR-Cas Type I-E
DRT Class 3
RM Type IIG
Argonaute Type III
Pycsar
Pycsar (other)
RM Type III
AbiE
RosmerTA
Zorya Type I
CRISPR-Cas Type I-F1
CBASS Type III
Wadjet Type I
BrxU
RM Type I
PD-T4-6
SoFic
IetAS
CBASS Type II
Hachiman Type I
CBASS Type I
Druantia Type II
Shedu
Lamassu Family
Mokosh Type I
Retron III-A
Shango
Gabija
PD-T4-7
AbiU
RM Type II
PD-T7-3
GAO 19
CBASS (other)
DUF4238
PD-Lambda-5
CRISPR-Cas Type I-C
0.0
0.5
1.0
1.5
2.0
Fraction
b.
Mom
AcrIC4
AcrIF6
AcrIF11
AcrIC3
AcrIF2 c2
AcrIE2
AcrIE3
Ardb Klca AcrIC11
Vcrx091_093
AcrIF7
AcrIF23
Ardc
AcrIF5
AcrIF24
Ardk
Arda
Acb1
Adnd
Aca alone
Acb2
AcrIF3
AcrIE1
AcrIF12
0.0
0.5
1.0
1.5
2.0
Fraction
GI-Hotspot 1
GI-Hotspot 2
GI-Hotspot 3
Elsewhere

Supplementary Figure 2. Distribution of defence and anti-defence systems between GI-Hotspot 1,2,3 and the rest of the genome. (a–b) Stacked bar plots show the relative contribution of GI-Hotspot 1,2,3 compared with the remainder of the genome ("Elsewhere") for the top 50 defence systems **(a)** and anti-defence systems **(b),** ranked by total count. Bars represent fractional contributions.

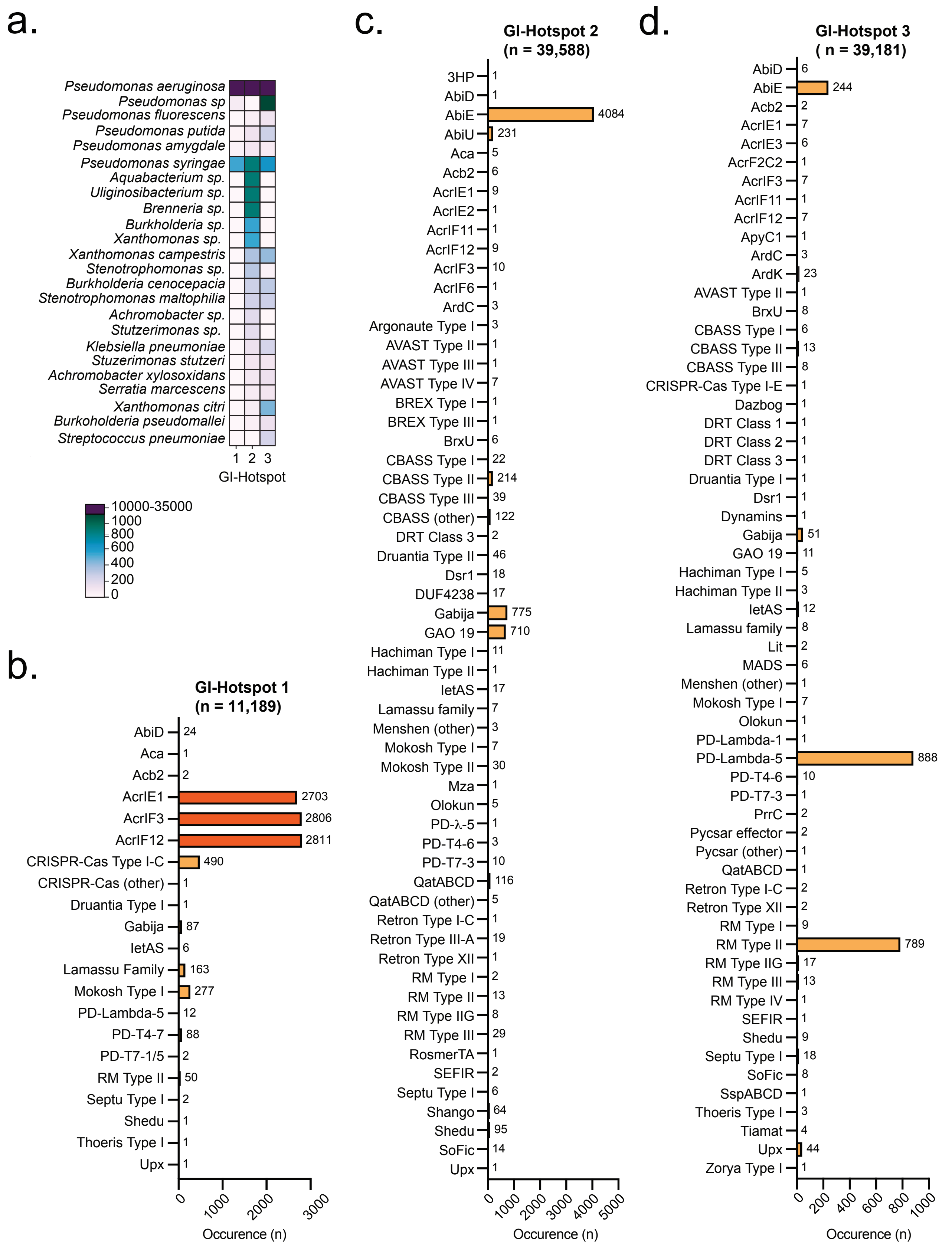
a.
Pseudomonas aeruginosa
Pseudomonas sp
Pseudomonas fluorescens
Pseudomonas putida
Pseudomonas amygdale
Pseudomonas syringae
Aquabacterium sp.
Uliginosibacterium sp.
Brenneria sp.
Burkholderia sp.
Xanthomonas sp.
Xanthomonas campestris
Stenotrophomonas sp.
Burkholderia cenocepacia
Stenotrophomonas maltophilia
Achromobacter sp.
Stutzerimonas sp.
Klebsiella pneumoniae
Stuzerimonas stutzeri
Achromobacter xylosoxidans
Serratia marcescens
Xanthomonas citri
Burkoholderia pseudomallei
Streptococcus pneumoniae
1 2 3
GI-Hotspot
10000-35000
1000
800
600
400
200
0
b.
GI-Hotspot 1
(n = 11,189)
AbiD 24
Aca 1
Acb2 2
AcrIE1 2703
AcrIF3 2806
AcrIF12 2811
CRISPR-Cas Type I-C 490
CRISPR-Cas (other) 1
Druantia Type I 1
Gabija 87
IetAS 6
Lamassu Family 163
Mokosh Type I 277
PD-Lambda-5 12
PD-T4-7 88
PD-T7-1/5 2
RM Type II 50
Septu Type I 2
Shedu 1
Thoeris Type I 1
Upx 1
0 1000 2000 3000
Occurence (n)
c.
GI-Hotspot 2
(n = 39,588)
3HP 1
AbiD 1
AbiE 4084
AbiU 231
Aca 5
Acb2 6
AcrIE1 9
AcrIE2 1
AcrIF11 1
AcrIF12 9
AcrIF3 10
AcrIF6 1
ArdC 3
Argonaute Type I 3
AVAST Type II 1
AVAST Type III 1
AVAST Type IV 7
BREX Type I 1
BREX Type III 1
BrxU 6
CBASS Type I 22
CBASS Type II 214
CBASS Type III 39
CBASS (other) 122
DRT Class 3 2
Druantia Type II 46
Dsr1 18
DUF4238 17
Gabija 775
GAO 19 710
Hachiman Type I 11
Hachiman Type II 1
IetAS 17
Lamassu family 7
Menshen (other) 3
Mokosh Type I 7
Mokosh Type II 30
Mza 1
Olokun 5
PD-λ-5 1
PD-T4-6 3
PD-T7-3 10
QatABCD 116
QatABCD (other) 5
Retron Type I-C 1
Retron Type III-A 19
Retron Type XII 1
RM Type I 2
RM Type II 13
RM Type IIG 8
RM Type III 29
RosmerTA 1
SEFIR 2
Septu Type I 6
Shango 64
Shedu 95
SoFic 14
Upx 1
0 1000 2000 3000 4000 5000
Occurence (n)
d.
GI-Hotspot 3
(n = 39,181)
AbiD 6
AbiE 244
Acb2 2
AcrIE1 7
AcrIE3 6
AcrF2C2 1
AcrIF3 7
AcrIF11 1
AcrIF12 7
ApyC1 1
ArdC 3
ArdK 23
AVAST Type II 1
BrxU 8
CBASS Type I 6
CBASS Type II 13
CBASS Type III 8
CRISPR-Cas Type I-E 1
Dazbog 1
DRT Class 1 1
DRT Class 2 1
DRT Class 3 1
Druantia Type I 1
Dsr1 1
Dynamins 1
Gabija 51
GAO 19 11
Hachiman Type I 5
Hachiman Type II 3
IetAS 12
Lamassu family 8
Lit 2
MADS 6
Menshen (other) 1
Mokosh Type I 7
Olokun 1
PD-Lambda-1 1
PD-Lambda-5 888
PD-T4-6 10
PD-T7-3 1
PrrC 2
Pycsar effector 2
Pycsar (other) 1
QatABCD 1
Retron Type I-C 2
Retron Type XII 2
RM Type I 9
RM Type II 789
RM Type IIG 17
RM Type III 13
RM Type IV 1
SEFIR 1
Shedu 9
Septu Type I 18
SoFic 8
SspABCD 1
Thoeris Type I 3
Tiamat 4
Upx 44
Zorya Type I 1
0 200 400 600 800 1000
Occurence (n)

Supplementary Figure 3. GI-Hotspot 1,2 and 3 are present in other genomic contexts. (a) Heatmap showing the abundance of GI-Hotspot 1, Hotspot 2, Hotspot 3 across bacterial species. Only species with at least 100 instances of any hotspot are shown. Counts were normalised by collapsing values between 10,000 and 35,000 to a fixed threshold of 20,000 to avoid visual distortion from outliers. Hotspot abundances are represented on a colour scale as showed in in figure legend. **(b-d)** Barplot showing the prevalence of defence and anti-defence systems found in GI-Hotspot 1 **(b)** in GI-Hotspot 2 **(c)** and in GI-Hotspot 3 **(d)** encoded on genomic contexts identified through cblaster (defence predicted with PADLOC). For panels **b-d,** bar plots show the number of occurrences of each defence or anti-defence system within GI-Hotspot 1,2,3 when identified through cblaster. The total number of hotspot instances (n) is indicated above each bar chart. Dataset sizes differ, so absolute proportions should not be directly compared across hotspots. Anti-defence systems are depicted in bright orange and defence systems in pale orange.

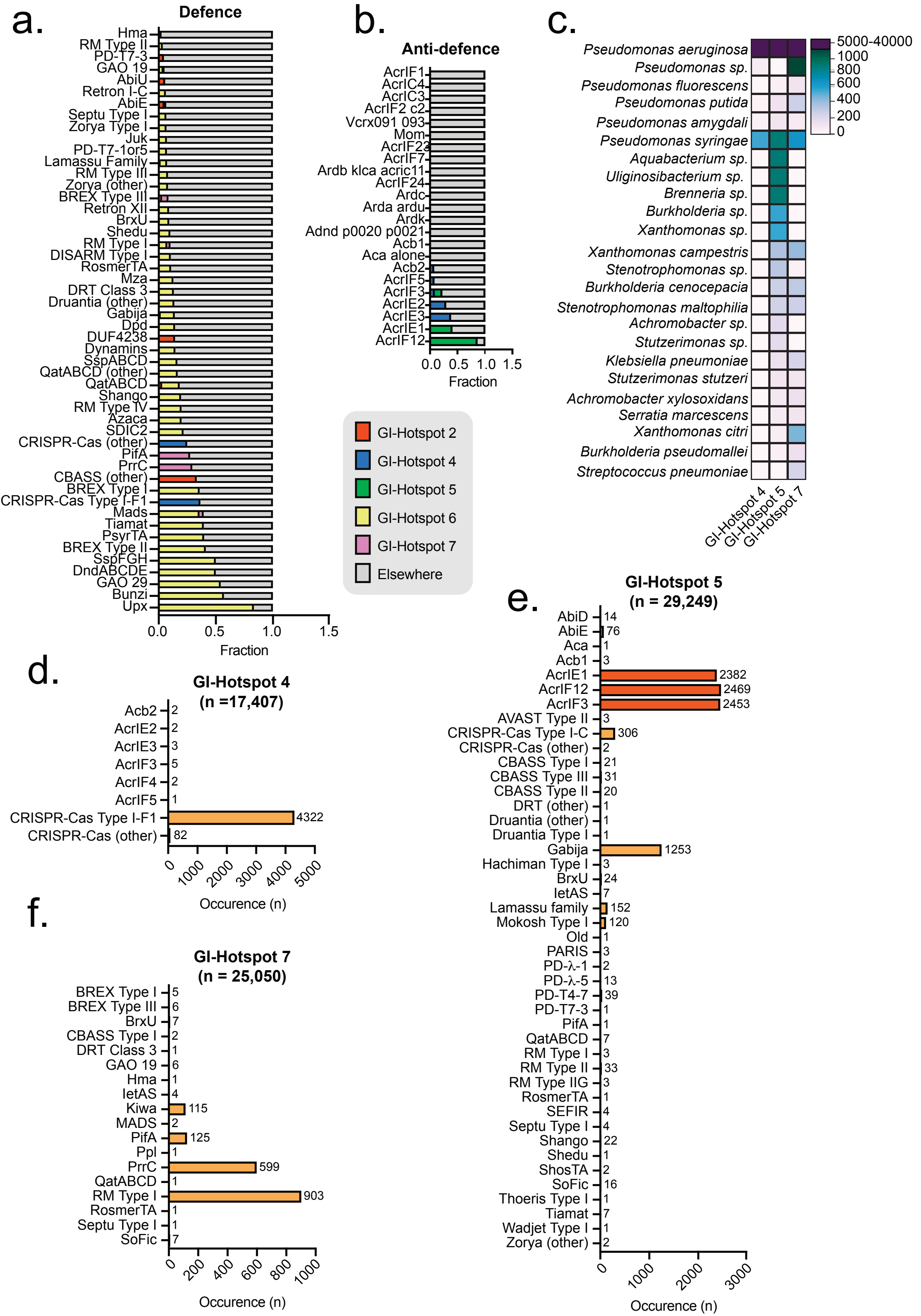
a.
Defence
Hma
RM Type II
PD-T7-3
GAO 19
AbiU
Retron I-C
AbiE
Septu Type I
Zorya Type I
Juk
PD-T7-1or5
Lamassu Family
RM Type III
Zorya (other)
BREX Type III
Retron XII
BrxU
Shedu
RM Type I
DISARM Type I
RosmerTA
Mza
DRT Class 3
Druantia (other)
Gabija
Dpd
DUF4238
Dynamins
SspABCD
QatABCD (other)
QatABCD
Shango
RM Type IV
Azaca
SDIC2
CRISPR-Cas (other)
PifA
PrrC
CBASS (other)
BREX Type I
CRISPR-Cas Type I-F1
Mads
Tiamat
PsyrTA
BREX Type II
SspFGH
DndABCDE
GAO 29
Bunzi
Upx
0.0 0.5 1.0 1.5
Fraction
b.
Anti-defence
AcrIF1
AcrIC4
AcrIC3
AcrIF2 c2
Vcrx091 093
Mom
AcrIF23
AcrIF7
Ardb klca acric11
AcrIF24
Ardc
Arda ardu
Ardk
Adnd p0020 p0021
Acb1
Aca alone
Acb2
AcrIF5
AcrIF3
AcrIE2
AcrIE3
AcrIE1
AcrIF12
0.0 0.5 1.0 1.5
Fraction
GI-Hotspot 2
GI-Hotspot 4
GI-Hotspot 5
GI-Hotspot 6
GI-Hotspot 7
Elsewhere
c.
Pseudomonas aeruginosa
Pseudomonas sp.
Pseudomonas fluorescens
Pseudomonas putida
Pseudomonas amygdali
Pseudomonas syringae
Aquabacterium sp.
Uliginosibacterium sp.
Brenneria sp.
Burkholderia sp.
Xanthomonas sp.
Xanthomonas campestris
Stenotrophomonas sp.
Burkholderia cenocepacia
Stenotrophomonas maltophilia
Achromobacter sp.
Stutzerimonas sp.
Klebsiella pneumoniae
Stutzerimonas stutzeri
Achromobacter xylosoxidans
Serratia marcescens
Xanthomonas citri
Burkholderia pseudomallei
Streptococcus pneumoniae
GI-Hotspot 4
GI-Hotspot 5
GI-Hotspot 7
5000-40000
1000
800
600
400
200
0
d.
GI-Hotspot 4
(n =17,407)
Acb2 2
AcrIE2 2
AcrIE3 3
AcrIF3 5
AcrIF4 2
AcrIF5 1
CRISPR-Cas Type I-F1 4322
CRISPR-Cas (other) 82
0 1000 2000 3000 4000 5000
Occurence (n)
e.
GI-Hotspot 5
(n = 29,249)
AbiD 14
AbiE 76
Aca 1
Acb1 3
AcrIE1 2382
AcrIF12 2469
AcrIF3 2453
AVAST Type II 3
CRISPR-Cas Type I-C 306
CRISPR-Cas (other) 2
CBASS Type I 21
CBASS Type III 31
CBASS Type II 20
DRT (other) 1
Druantia (other) 1
Druantia Type I 1
Gabija 1253
Hachiman Type I 3
BrxU 24
IetAS 7
Lamassu family 152
Mokosh Type I 120
Old 1
PARIS 3
PD-λ-1 2
PD-λ-5 13
PD-T4-7 39
PD-T7-3 1
PifA 1
QatABCD 7
RM Type I 3
RM Type II 33
RM Type IIG 3
RosmerTA 1
SEFIR 4
Septu Type I 4
Shango 22
Shedu 1
ShosTA 2
SoFic 16
Thoeris Type I 1
Tiamat 7
Wadjet Type I 1
Zorya (other) 2
0 1000 2000 3000
Occurence (n)
f.
GI-Hotspot 7
(n = 25,050)
BREX Type I 5
BREX Type III 6
BrxU 7
CBASS Type I 2
DRT Class 3 1
GAO 19 6
Hma 1
IetAS 4
Kiwa 115
MADS 2
PifA 125
Ppl 1
PrrC 599
QatABCD 1
RM Type I 903
RosmerTA 1
Septu Type I 1
SoFic 7
0 200 400 600 800 1000
Occurence (n)

Supplementary Figure 4. GI-Hotspot 4,5,7 are conserved in other genomic contexts.(a-b) Horizontal stacked bar plots showing the relative contribution of GI-Hotspot 2,4,5,6,7 compared with the remainder of the genome ("Elsewhere") for the top 50 defence systems **(a)** and anti-defence systems **(b),** ranked by total count. Bars represent fractional contributions. **(c)** Heatmap showing the abundance of GI-Hotspot 4, 5 and 7 across bacterial species. Only species with at least 100 instances of any hotspot are shown. Counts were normalised by collapsing values between 10,000 and 35,000 to a fixed threshold of 20,000 to avoid visual distortion. Hotspot abundances are represented on a colour scale as showed in in figure legend. **(d-f)** Barplot showing the prevalence of defence and anti-defence systems found in GI-Hotspot 4 **(d)**, GI-Hotspot 5 **(e)** and GI-Hotspot 7 **(f)** encoded on genomic contexts identified through cblaster (defence predicted with PADLOC). For panels **d–f,** bar plots show the number of occurrences of each defence or anti-defence system within GI-Hotspot 4,5,7 when identified through cblaster. The total number of hotspot instances (n) is indicated above each bar chart. Dataset sizes differ, so absolute proportions should not be directly compared across hotspots. Anti-defence systems are depicted in bright orange and defence systems in pale orange.

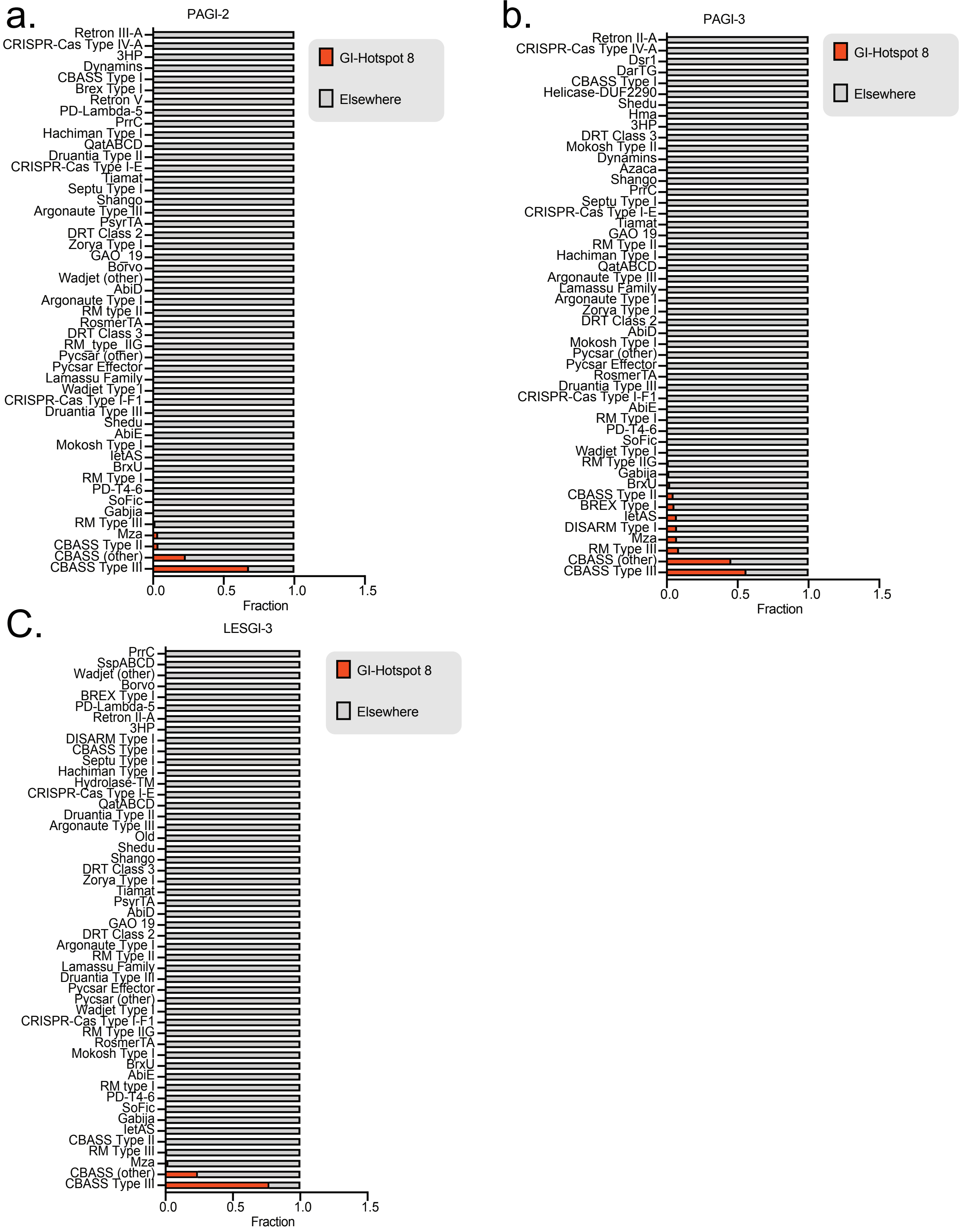

a.
PAGI-2
Retron III-A
CRISPR-Cas Type IV-A
3HP
Dynamins
CBASS Type I
Brex Type I
Retron V
PD-Lambda-5
PrrC
Hachiman Type I
QatABCD
Druantia Type II
CRISPR-Cas Type I-E
Tiamat
Septu Type I
Shango
Argonaute Type III
PsyrTA
DRT Class 2
Zorya Type I
GAO_19
Borvo
Wadjet (other)
AbiD
Argonaute Type I
RM type II
RosmerTA
DRT Class 3
RM_type_IIG
Pycsar (other)
Pycsar Effector
Lamassu Family
Wadjet Type I
CRISPR-Cas Type I-F1
Druantia Type III
Shedu
AbiE
Mokosh Type I
IetAS
BrxU
RM Type I
PD-T4-6
SoFic
Gabija
RM Type III
Mza
CBASS Type II
CBASS (other)
CBASS Type III
0.0
0.5
1.0
1.5
Fraction
GI-Hotspot 8
Elsewhere
b.
PAGI-3
Retron II-A
CRISPR-Cas Type IV-A
Dsr1
DarTG
CBASS Type I
Helicase-DUF2290
Shedu
Hma
3HP
DRT Class 3
Mokosh Type II
Dynamins
Azaca
Shango
PrrC
Septu Type I
CRISPR-Cas Type I-E
Tiamat
GAO 19
RM Type II
Hachiman Type I
QatABCD
Argonaute Type III
Lamassu Family
Argonaute Type I
Zorya Type I
DRT Class 2
AbiD
Mokosh Type I
Pycsar (other)
Pycsar Effector
RosmerTA
Druantia Type III
CRISPR-Cas Type I-F1
AbiE
RM Type I
PD-T4-6
SoFic
Wadjet Type I
RM Type IIG
Gabija
BrxU
CBASS Type II
BREX Type I
IetAS
DISARM Type I
Mza
RM Type III
CBASS (other)
CBASS Type III
0.0
0.5
1.0
1.5
Fraction
GI-Hotspot 8
Elsewhere
c.
LESGI-3
PrrC
SspABCD
Wadjet (other)
Borvo
BREX Type I
PD-Lambda-5
Retron II-A
3HP
DISARM Type I
CBASS Type I
Septu Type I
Hachiman Type I
Hydrolase-TM
CRISPR-Cas Type I-E
QatABCD
Druantia Type II
Argonaute Type III
Old
Shedu
Shango
DRT Class 3
Zorya Type I
Tiamat
PsyrTA
AbiD
GAO 19
DRT Class 2
Argonaute Type I
RM Type II
Lamassu Family
Druantia Type III
Pycsar Effector
Pycsar (other)
Wadjet Type I
CRISPR-Cas Type I-F1
RM Type IIG
RosmerTA
Mokosh Type I
BrxU
AbiE
RM type I
PD-T4-6
SoFic
Gabija
IetAS
CBASS Type II
RM Type III
Mza
CBASS (other)
CBASS Type III
0.0
0.5
1.0
1.5
Fraction
GI-Hotspot 8
Elsewhere

Supplementary Figure 5. Distribution of defence and anti-defence systems between GI-Hotspot 8 and the rest of the genome. (a–c) Horizontal stacked bar plots show the relative contribution of GI-Hotspot 8 compared with the remainder of the genome ("Elsewhere") for the top 50 defence systems in PAGI-2 **(a),** PAGI-3 **(b),** and LESGI-3 **(c).** Bars represent fractional contributions.

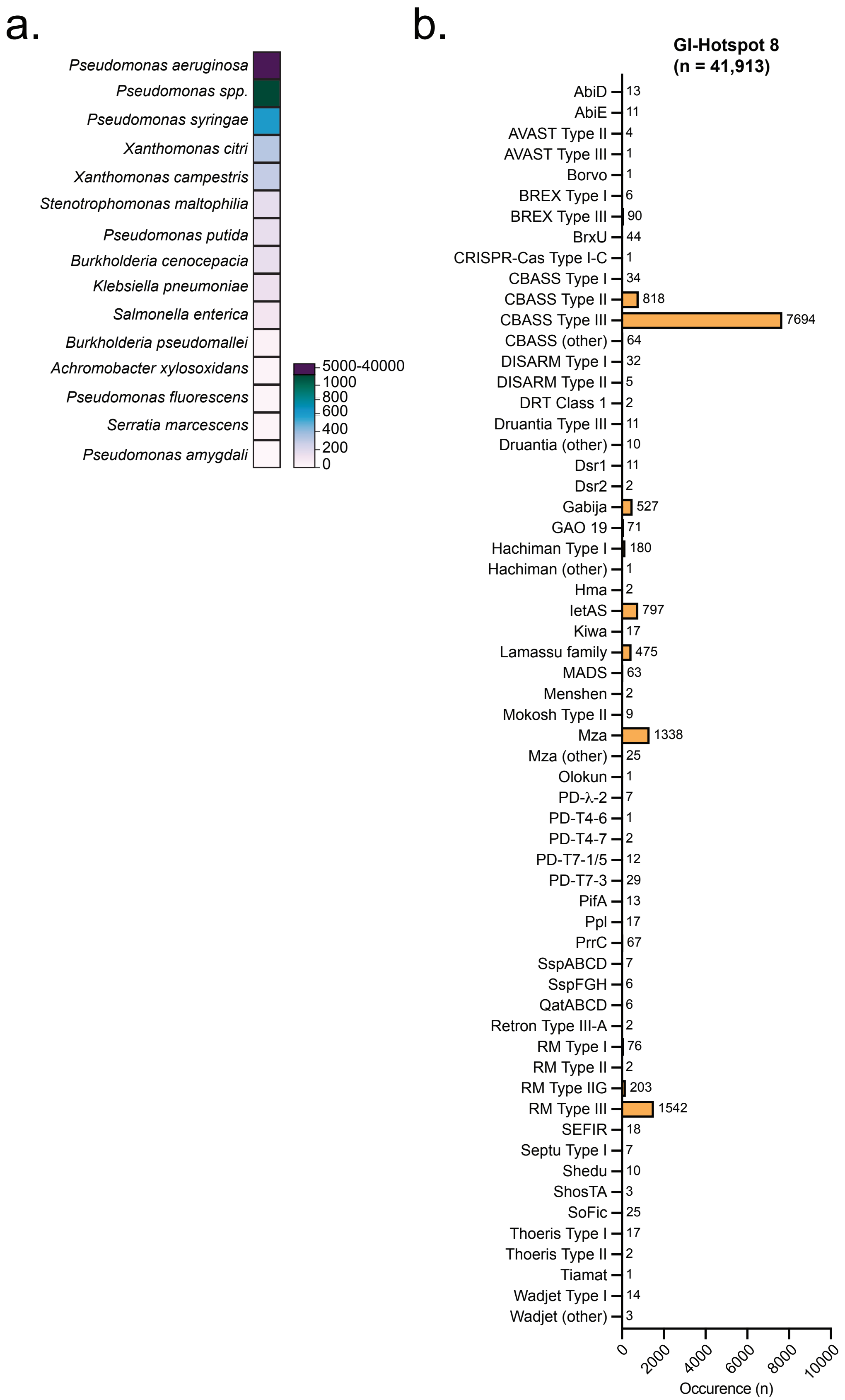
a.
Pseudomonas aeruginosa
Pseudomonas spp.
Pseudomonas syringae
Xanthomonas citri
Xanthomonas campestris
Stenotrophomonas maltophilia
Pseudomonas putida
Burkholderia cenocepacia
Klebsiella pneumoniae
Salmonella enterica
Burkholderia pseudomallei
Achromobacter xylosoxidans
Pseudomonas fluorescens
Serratia marcescens
Pseudomonas amygdali
5000-40000
1000
800
600
400
200
0
b.
GI-Hotspot 8
(n = 41,913)
AbiD 13
AbiE 11
AVAST Type II 4
AVAST Type III 1
Borvo 1
BREX Type I 6
BREX Type III 90
BrxU 44
CRISPR-Cas Type I-C 1
CBASS Type I 34
CBASS Type II 818
CBASS Type III 7694
CBASS (other) 64
DISARM Type I 32
DISARM Type II 5
DRT Class 1 2
Druantia Type III 11
Druantia (other) 10
Dsr1 11
Dsr2 2
Gabija 527
GAO 19 71
Hachiman Type I 180
Hachiman (other) 1
Hma 2
IetAS 797
Kiwa 17
Lamassu family 475
MADS 63
Menshen 2
Mokosh Type II 9
Mza 1338
Mza (other) 25
Olokun 1
PD-λ-2 7
PD-T4-6 1
PD-T4-7 2
PD-T7-1/5 12
PD-T7-3 29
PifA 13
Ppl 17
PrrC 67
SspABCD 7
SspFGH 6
QatABCD 6
Retron Type III-A 2
RM Type I 76
RM Type II 2
RM Type IIG 203
RM Type III 1542
SEFIR 18
Septu Type I 7
Shedu 10
ShosTA 3
SoFic 25
Thoeris Type I 17
Thoeris Type II 2
Tiamat 1
Wadjet Type I 14
Wadjet (other) 3
0
2000
4000
6000
8000
10000
Occurence (n)

Supplementary Figure 6. GI-Hotspot 8 is widespread in *Pseudomonas* and other species. (a) Heatmap showing the abundance of GI-Hotspot 8 across bacterial species. Only species with at least 100 instances of any hotspot are shown. Counts were normalised by collapsing values between 10,000 and 35,000 to a fixed threshold of 20,000 to avoid visual distortion from outliers. Hotspot abundances are represented on a colour scale as showed in in figure legend. **(b)** Barplot showing the prevalence of defence and anti-defence systems found in GI-Hotspot 8 when identified through cblaster searches. For panel **b,** bar plots show the number of occurrences of each defence system within GI-Hotspot 8 when identified through cblaster (defence predicted with PADLOC). The total number of hotspot instances (n) is indicated above each bar chart. Dataset sizes differ, so absolute proportions should not be directly compared across hotspots. Anti-defence systems are depicted in bright orange and defence systems in pale orange.

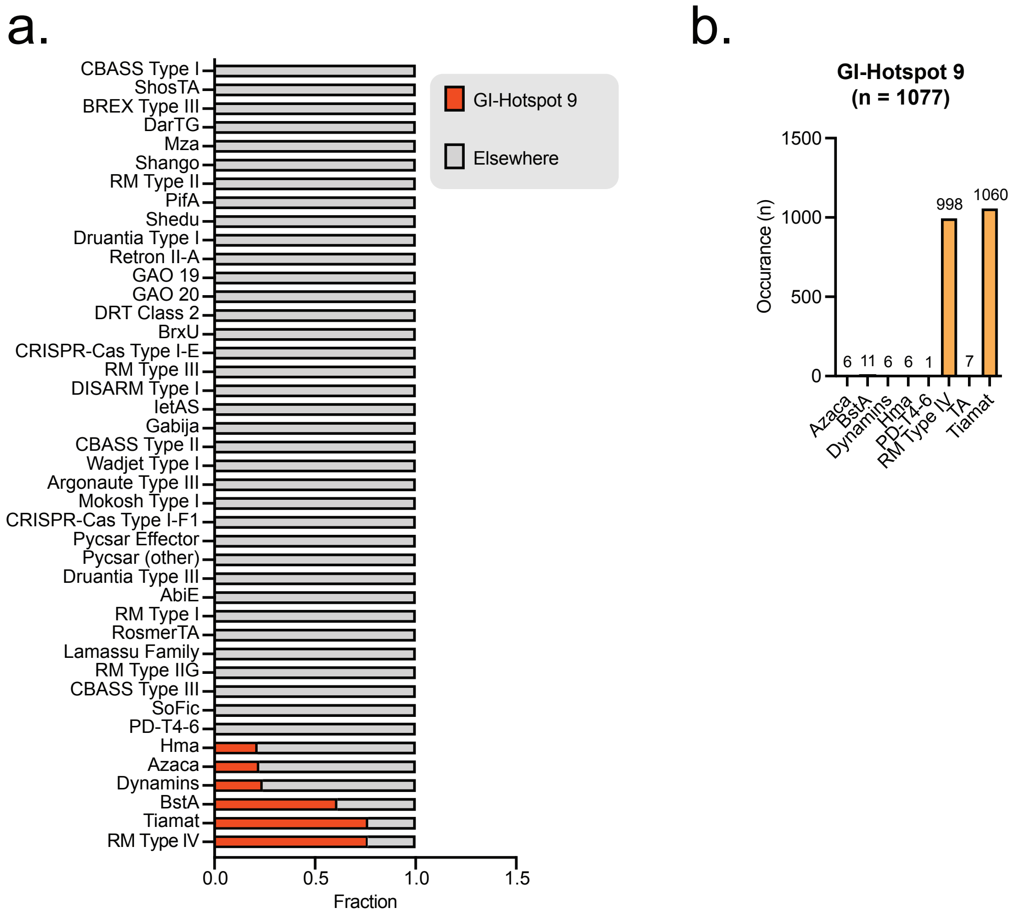

a.
CBASS Type I
ShosTA
BREX Type III
DarTG
Mza
Shango
RM Type II
PifA
Shedu
Druantia Type I
Retron II-A
GAO 19
GAO 20
DRT Class 2
BrxU
CRISPR-Cas Type I-E
RM Type III
DISARM Type I
IetAS
Gabija
CBASS Type II
Wadjet Type I
Argonaute Type III
Mokosh Type I
CRISPR-Cas Type I-F1
Pycsar Effector
Pycsar (other)
Druantia Type III
AbiE
RM Type I
RosmerTA
Lamassu Family
RM Type IIG
CBASS Type III
SoFic
PD-T4-6
Hma
Azaca
Dynamins
BstA
Tiamat
RM Type IV
0.0
0.5
1.0
1.5
Fraction
GI-Hotspot 9
Elsewhere
b.
GI-Hotspot 9
(n = 1077)
Occurance (n)
1500
1000
500
0
6
11
6
6
1
998
7
1060
Azaca
BstA
Dynamins
Hma
PD-T4-6
RM Type IV
TA
Tiamat

Supplementary Figure 7. LESGI-3 GI-Hotspot 9 preferentially carries Tiamat. (a) Horizontal stacked bar plots show the relative contribution of GI-Hotspot 9 compared with the remainder of the genome (“Elsewhere”) for the top 50 defence systems ranked by total count. Bars represent fractional contributions. **(b)** Barplot showing the prevalence of defence and anti-defence systems found in GI-Hotspot 9 when identified through cblaster searches (defence predicted with PADLOC). For panel **b,** bar plots show the number of occurrences of each defence system within GI-Hotspot 9 when identified through cblaster. The total number of hotspot instances (n) is indicated above each bar chart. Dataset sizes differ, so absolute proportions should not be directly compared across hotspots. Anti-defence systems are depicted in bright orange and defence systems in pale orange.

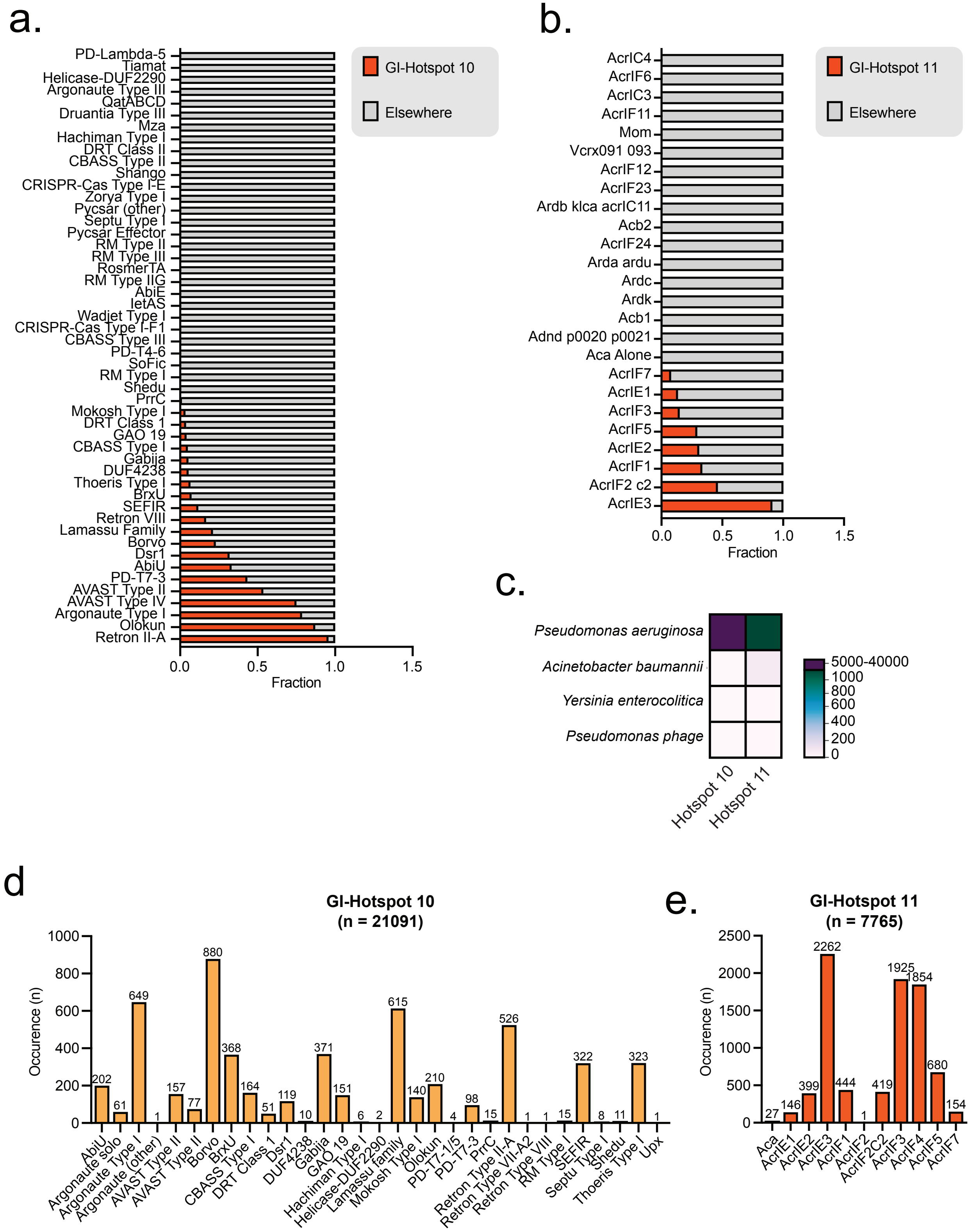
a.
PD-Lambda-5
Tiamat
Helicase-DUF2290
Argonaute Type III
QatABCD
Druantia Type III
Mza
Hachiman Type I
DRT Class II
CBASS Type II
Shango
CRISPR-Cas Type I-E
Zorya Type I
Pycsar (other)
Septu Type I
Pycsar Effector
RM Type II
RM Type III
RosmerTA
RM Type IIG
AbiE
IetAS
Wadjet Type I
CRISPR-Cas Type I-F1
CBASS Type III
PD-T4-6
SoFic
RM Type I
Shedu
PrrC
Mokosh Type I
DRT Class 1
GAO 19
CBASS Type I
Gabija
DUF4238
Thoeris Type I
BrxU
SEFIR
Retron VIII
Lamassu Family
Borvo
Dsr1
AbiU
PD-T7-3
AVAST Type II
AVAST Type IV
Argonaute Type I
Olokun
Retron II-A
0.0
0.5
1.0
1.5
Fraction
GI-Hotspot 10
Elsewhere
b.
AcrIC4
AcrIF6
AcrIC3
AcrIF11
Mom
Vcrx091 093
AcrIF12
AcrIF23
Ardb klca acrIC11
Acb2
AcrIF24
Arda ardu
Ardc
Ardk
Acb1
Adnd p0020 p0021
Aca Alone
AcrIF7
AcrIE1
AcrIF3
AcrIF5
AcrIE2
AcrIF1
AcrIF2 c2
AcrIE3
0.0
0.5
1.0
1.5
Fraction
GI-Hotspot 11
Elsewhere
c.
Pseudomonas aeruginosa
Acinetobacter baumannii
Yersinia enterocolitica
Pseudomonas phage
Hotspot 10
Hotspot 11
5000-40000
1000
800
600
400
200
0
d
GI-Hotspot 10
(n = 21091)
Occurence (n)
1000
800
600
400
200
0
202
61
649
1
157
77
880
368
164
51
119
10
371
151
6
2
615
140
210
4
98
15
526
1
1
15
322
8
11
323
1
AbiU
Argonaute solo
Argonaute Type I
Argonaute (other)
AVAST Type II
AVAST Type II
Borvo
BrxU
CBASS Type I
DRT Class 1
Dsr1
DUF4238
Gabija
GAO 19
Hachiman Type I
Helicase-DUF2290
Lamassu family
Mokosh Type I
Olokun
PD-T7-1/5
PD-T7-3
PrrC
Retron Type II-A
Retron Type VII-A2
Retron Type VIII
RM Type I
SEFIR
Septu Type I
Shedu
Thoeris Type I
Upx
e.
GI-Hotspot 11
(n = 7765)
Occurence (n)
2500
2000
1500
1000
500
0
27
146
399
2262
444
1
419
1925
1854
680
154
Aca
AcrIE1
AcrIE2
AcrIE3
AcrIF1
AcrIF2
AcrIF2C2
AcrIF3
AcrIF4
AcrIF5
AcrIF7

Supplementary Figure 8. GI-Hotspot 10 and 11 retain their functional specialisation in broader genomic contexts (a-b) Horizontal stacked bar plots show the relative contribution of GI-Hotspot 10 and 11 compared with the remainder of the genome ("Elsewhere") for the top 50 defence systems **(a)** and anti-defence systems **(b),** ranked by total count. Bars represent fractional contributions. **(c)** Heatmap showing the abundance of GI-Hotspot 10 and 11 across bacterial species. Only species with at least 100 instances of any hotspot are shown. Counts were normalised by collapsing values between 10,000 and 35,000 to a fixed threshold of 20,000 to avoid visual distortion. Hotspot abundances are represented on a colour scale as showed in in figure legend. **(d-e)** Barplot showing the prevalence of defence and anti-defence systems found in GI-Hotspot 10 **(d)** and GI-Hotspot 11 **(e)** encoded on genomic contexts identified through cblaster (defence predicted with PADLOC). For panel **d-e,** bar plots show the number of occurrences of each defence or anti-defence system within GI-Hotspot 10 and 11 when identified through cblaster. The total number of hotspot instances (n) is indicated above each bar chart. Dataset sizes differ, so absolute proportions should not be directly compared across hotspots. Anti-defence systems are depicted in bright orange and defence systems in pale orange.
